# Protein lysine 43 methylation by EZH1 promotes AML1-ETO transcriptional repression in leukemia

**DOI:** 10.1038/s41467-019-12960-6

**Published:** 2019-11-07

**Authors:** Liping Dou, Fei Yan, Jiuxia Pang, Dehua Zheng, Dandan Li, Li Gao, Lijun Wang, Yihan Xu, Jinlong Shi, Qian Wang, Lei Zhou, Na Shen, Puja Singh, Lili Wang, Yonghui Li, Yvchi Gao, Tao Liu, Chongjian Chen, Aref Al-Kali, Mark R. Litzow, Young-In Chi, Ann M. Bode, Chunhui Liu, Haojie Huang, Daihong Liu, Guido Marcucci, Shujun Liu, Li Yu

**Affiliations:** 1 Department of Hematology, Chinese PLA General Hospital, Medical School of Chinese PLA, 28 Fuxing Road, 100853 Beijing, China; 2 The Hormel Institute, University of Minnesota, 801 16th Avenue NE, Austin, MN 55912 USA; 3 State Key Laboratory of Inorganic Synthesis and Preparative Chemistry, International Joint Research Laboratory of Nano-Micro Architecture Chemistry (NMAC), International Research Center for Chemistry-Medicine Joint Innovation, College of Chemistry, Jilin University, 2699 Qianjin Street, 130012 Changchun, China; 4 Department of Hepatology, Chinese PLA General Hospital, Medical School of Chinese PLA, 28 Fuxing Road, 100853 Beijing, China; 5 Annoroad Gene Technical Laboratory, 6 Kechuang road, 100176 Beijing, China; 6 Division of Hematology, Mayo Clinic, 200 1st Street SW, Rochester, MN 55905 USA; 7 Haoshi Biotechnical Laboratory, 1 Pingshan First Road, 518055 Shenzhen, China; 8 Department of Biochemistry and Molecular Biology, Mayo Clinic, 200 1st Street SW, Rochester, MN 55905 USA; 9 Department of Hematologic Malignancies Translational Science, City of Hope, 1500 East Duarte Road, Duarte, CA 91010 USA; 10 Department of Hematology-Oncology, International Cancer Center, Shenzhen University General Hospital, Shenzhen University Health Science Center, 1098 Xueyuan Ave, 518060 Shenzhen, China

**Keywords:** Acute myeloid leukaemia, Molecular biology, Methylation, Transcription, Leukaemia

## Abstract

The oncogenic fusion protein AML1-ETO retains the ability of AML1 to interact with the enhancer core DNA sequences, but blocks AML1-dependent transcription. Previous studies have shown that post-translational modification of AML1-ETO may play a role in its regulation. Here we report that AML1-ETO-positive patients, with high histone lysine methyltransferase Enhancer of zeste homolog 1 (EZH1) expression, show a worse overall survival than those with lower EZH1 expression. EZH1 knockdown impairs survival and proliferation of AML1-ETO-expressing cells in vitro and in vivo. We find that EZH1 WD domain binds to the AML1-ETO NHR1 domain and methylates AML1-ETO at lysine 43 (Lys43). This requires the EZH1 SET domain, which augments AML1-ETO-dependent repression of tumor suppressor genes. Loss of Lys43 methylation by point mutation or domain deletion impairs AML1-ETO-repressive activity. These findings highlight the role of EZH1 in non-histone lysine methylation, indicating that cooperation between AML1-ETO and EZH1 and AML1-ETO site-specific lysine methylation promote AML1-ETO transcriptional repression in leukemia.

## Abstract (web summary)

The oncogenic fusion protein AML1-ETO has the ability of AML1 to interact with DNA but blocks AML1-dependent transcription. Here the authors report that histone lysine methyltransferase EZH1 interacts with AML1-ETO and methylates AML1-ETO at lysine 43, promoting AML1-ETO transcriptional repression in leukemia.

## Introduction

The fusion protein AML1-ETO, which originates from the t(8;21)(q22;q22) chromosomal translocation, is found in ~12% of adults with acute myeloid leukemia (AML). AML1-ETO retains the ability of AML1 to interact with the enhancer core DNA sequence, but blocks AML1-dependent transcription^1–4^. Although it activates certain genes^5^, AML1-ETO dominantly represses AML1 targets^1,5^, leading to perturbation of normal hematopoiesis. Mechanistically, AML1-ETO recruits co-partners (e.g., HDACs, DNMTs, and p300)^5–8^ to target promoters resulting in gene repression/activation. It still remains largely unknown how AML1-ETO interacts with diverse transcriptional factors and target DNAs. Previous studies showed that p300 acetylates AML1-ETO at lysine 43 (Lys43) thus enhancing AML1-ETO functions^5^. Treatment with p300 inhibitors decreases AML1-ETO acetylation leading to a blockage of AML progression. However, the facts that the AML1 Lys43 acetylation is not fully in line with its DNA binding affinity and transcriptional responses^9^, and that the p300 inactivation only partially impairs AML1-ETO functions^5^, suggest the existence of additional interacting proteins/modifications that fine-tune AML1-ETO leukemogenic activities.

Although observed less frequently, lysine methylation of non-histone proteins (e.g., p53^10^, TAF10^11^, RORA^12^, GATA4^13^, STAT3^14^) critically regulates their functions, and is essential for tumorigenesis^15^. AML1-ETO is a leukemia-initiating transcriptional factor that has many lysine residues. Yet whether and how AML1-ETO is subjected to regulation by lysine methylation is unclear. It is postulated that histone lysine methyltransferases, such as Enhancer of zeste homolog 2 (EZH2), G9a, SET7/8, and SETD6, account for non-histone protein lysine methylation^11,13,16,17^. EZH2 and its close homolog EZH1 are the catalytic subunits of the polycomb repressive complex 2 (PRC2), which compacts chromatin^18,19^ through histone H3 Lys27 methylation (H3K27me2/3)^20,21^. However, EZH1 and EZH2 are located in different PRC2 complexes, and EZH1 complements EZH2 in maintaining stem cell identity^22–25^. In addition, EZH1 possesses lower histone lysine methyltransferase activity compared to EZH2^26^, which is further supported by a recent study showing that EZH1 knockdown in mice has a limited effect on global trimethylation of H3K27^27^. These findings support the idea that EZH1 could regulate genes in a histone methylation-independent manner^25^, which has not been explored. In this study, we demonstrate that Lys43 methylation by EZH1 is critical means of AML1-ETO-driven transcription.

## Results

### EZH1 upregulation predicts worse prognosis in AML1-ETO AML

**Fig. 1:**
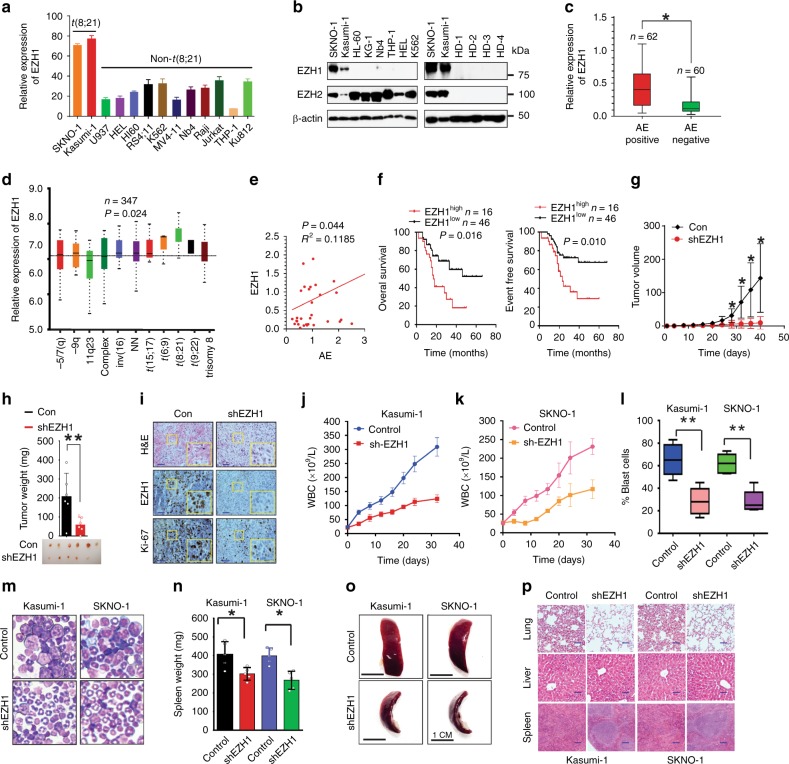
EZH1 promotes AML1-ETO leukemia. **a** qPCR for EZH1 mRNA expression in leukemia cell lines (*n* = 3). **b** Western blotting for EZH1 and EZH2 protein expression in leukemia cells lines and healthy human donors (HD). **c** qPCR for EZH1 mRNA expression in AML1-ETO-positive (*n* = 62) and negative (*n* = 60) leukemia patients. Median values are depicted by the horizontal lines. *P *<* 0.05. **d** Normalized EZH1 expression in primary cells of de novo AML patients (GSE6891) (*n* = 347). NN refers to normal karyotype. Patient characteristics are summarized in Supplementary Table 2. **e** Correlation analysis for AML1-ETO and EZH1 mRNA expression in AML1-ETO-positive leukemia patients (*n* = 62). R: Pearson correlation coefficients; R^2^: indicates “the goodness of fit”. Statistical significance was calculated by Pearson correlation coefficients. **f** The association of EZH1 expression with overall survival (OS) and event free survival (EFS) in AML1-ETO-positive patients (*n* = 62; from Fig. 1c) analyzed by the Kaplan–Meier estimate (*n* = 62). **g**, **h** EZH1-depleted and control SKNO-1 cells were injected subcutaneously into the flanks of nude mice. The measurement of xenograft tumor size (**g**), and the visual analysis and tumor weight (**h**) (*n* = 6 tumors/group). Data are expressed as mean values ± S.D. *P *<* 0.05, **P *<* 0.01. **i** Representative images of H&E-stained and IHC-stained tumor sections (original magnification ×200). Scale bar, 100 µm. Insets show high-magnification images of the boxed areas. **j**–**p** Kasumi-1 and SKNO-1 cells (1–3 × 10^6^) infected with EZH1 viruses were injected into NOD/SCID/γ_c_^null^ immunodeficient mice through the tail-vein (*n* = 5 mice/group). WBC analysis of peripheral blood (PB) (**j**, **k**), blast cells percentage in PB (**l**), Wright/Giemsa staining of bone marrow (**m**), graph illustration of the spleen weights (**n**), representative external views of the spleens (**o**) and H&E staining of sections of spleens, livers and lungs (**p**). Scale bars represent 100 µm (blue). Fig. **a**, data are expressed as mean values ± S.E.M. of triplicate samples from three independent experiments; Fig. **n**, data are expressed as mean values ± S.D. *P *<* 0.05, **P *<* 0.01; Fig. **c**, Mann–Whitney *U*-tests; Figs. **d**, **g**, **h**, **i**, **n**, one-way ANOVA; Fig. **f**, Log-rank test; For box-whisker plots, box edges correspond to 25th and 75th percentiles, lines inside the box correspond to 50th percentiles, and whiskers include extreme data points. AE AML1-ETO. The data in Fig. **b** are representative of 3 independent experiments. Source data are included in the Source Data file

Although their functional abnormalities are strongly associated with many types of cancer^27^, whether or how EZH1/2 regulates AML1-ETO leukemia remains elusive. We first examined the expression of EZH1/2 in 122 patient-derived AML samples (Supplementary Table 1), and 13 leukemia cell lines. Compared to their negative counterparts and healthy donors, *EZH1* mRNA and protein expression is much higher in AML1-ETO-positive cell lines, SKNO-1 and Kasumi-1 (Fig. 1a, b), and patient primary cells (Fig. 1c). We also analyzed a public database GSE6891^28^ including 347 AML patients. Consistently, EZH1 was upregulated significantly (*P* = 0.024 by the nonparametric Mann–Whitney *U* test) in t(8;21) compared to other types of patients (Fig. 1d and Supplementary Table 2). We then examined the relationship of AML1-ETO and EZH1 expression in AML1-ETO-positive patients (*n* = 62), and found a positive correlation of AML1-ETO with EZH1 (Fig. 1e). Importantly, patients with a higher level of EZH1 showed a worse overall survival (OS, median = 23.0 vs. 46.0 months, *P* = 0.016) and event-free survival (EFS, median = 15.1 vs. 28.0 months, *P* = 0.010) than those with lower EZH1 expression (Fig. 1f and Supplementary Table 3 of Univariate and multivariate analyses for OS and EFS in 62 AML patients). Although barely detectable in healthy donors, EZH2 was upregulated in both AML1-ETO-positive and AML1-ETO-negative leukemia cells (see Fig. 1b), suggesting an AML1-ETO-independent mechanism. Importantly, the levels of EZH2 did not exhibit significant correlation with either AML1-ETO expression (Supplementary Fig. 1a) or the OS and EFS survival of AML1-ETO-positive patients (Supplementary Fig. 1b) and AML patients reported in GSE6891^28^ (Supplementary Fig. 1c). These findings support the idea that EZH1 is more essential than EZH2 for AML1-ETO leukemia.

To experimentally demonstrate the role of EZH1 in AML1-ETO AML, we knocked down EZH1 in SKNO-1 and Kasumi-1 cells naturally expressing AML1-ETO and EZH1 (Supplementary Fig. 1d, e). We found that EZH1 downregulation inhibits cell proliferation (Supplementary Fig. 1f) and impairs colony-forming potential (Supplementary Fig. 1g). Flow cytometry analysis did not reveal apparent changes in Annexin-FITC-stained cells, but detected a decrease in S-phase population in EZH1-depleted cells (Supplementary Fig. 1h). To determine this effect in vivo, we subcutaneously injected EZH1-depleted SKNO-1 cells (0.3 × 10^6^) into the flanks of nude mice. Although tumor incidence was only slightly different between shRNA (5/6, 85%) and control (6/6, 100%), we observed a different tumor latency, with tumors detectable at 24 days in the scrambled control, but 32 days in the shRNA group after injection. Tumors in the controls grew faster with larger final tumor volume than those in the shRNA group (Fig. 1g). Further, mice engrafted with shRNA-transfected cells had lower tumor weight (Fig. 1h), EZH1 downregulation in tumors and impaired rate in tumor formation (Fig. 1i; Supplementary Fig. 1i). To further demonstrate the clinical relevance of EZH1 in leukemia, orthotopic xenograft was performed in NOD/SCID/γ_c_^null^ immunodeficient NOG mice (*n* = 5 mice/group). Briefly, Kasumi-1 and SKNO-1 cells were infected with EZH1 shRNA or scramble virus. After confirming EZH1 downregulation (Supplementary Fig. 1j), about 1–3 × 10^6^ cells were injected into the irradiated NOG mice through the tail vein. We observed that the mice in scrambled control develop more aggressive leukemia than those in shRNA group, which was supported by higher white blood cell (WBC) counts (Fig. 1j, k), more blast cells in peripheral blood (Fig. 1l) and in bone marrow (Fig. 1m). The less aggressive leukemia growth observed in EZH1 knockdown compared to control mice was further demonstrated by smaller spleens (Fig. 1n, o) and less infiltration of leukemia cells into spleens, lungs, and livers (Fig. 1p). Based on these data, we conclude that EZH1 facilitates the survival and proliferation of AML1-ETO-expressing cells.

### The AML1-ETO is monomethylated at Lys43 in its N terminus

**Fig. 2:**
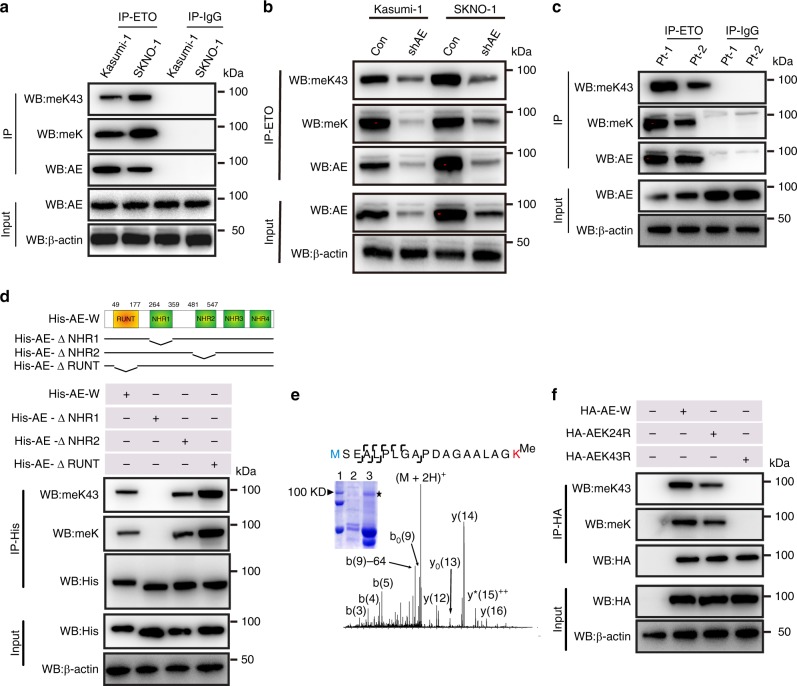
AML1-ETO is methylated at Lys43 in vitro and in vivo. **a** Western blotting for anti-ETO immunoprecipitates (lanes 1, 2) from Kasumi-1 and SKNO-1 cells. IgG pull-down performed in parallel (lanes 3, 4) was used as a negative control. **b** Kasumi-1 and SKNO-1 cells were transfected with AML1-ETO shRNA (shAE) for 48 h. The anti-ETO immunoprecipitates (IP) and total cell lysates (Input) were subjected to Western blotting. **c** Western blotting for anti-ETO immunoprecipitates (lanes 1, 2) from AML1-ETO-positive patient primary cells (*N* = 2). IgG pull-down performed in parallel (lanes 3, 4) was used as a negative control. Pt patient. **d** Upper: Diagram of AML1-ETO domain constructs; lower: Western blotting for anti-His immunoprecipitates from HEK293 cells expressing His-AE-W (wild type) (lane 1), His-AE-ΔNHR1 (lane 2), His-AE-ΔNHR2 (lane 3) or His-AE-ΔRUNT (lane 4). **e** The MS/MS fragmentation spectrum and pattern of the isolated peptide MSEALPLGAPDAG AALAGK. AML1-ETO was immunoprecipitated by anti-ETO from Kasumi-1 cells. (Inset) SDS-PAGE of precipitated proteins confirmed AML1-ETO pulldown (Coomassie blue-stained) in Kasumi-1 (lane 2) and HEK293 cells (lane 3). Lane 1 is the protein ladder marker. The AML1-ETO bands from the anti-ETO IP (indicated by star) were subjected to LC/MS/MS. The b-type and y-type product ions are marked in the spectrum. A 14 Da increase in the Lys43 residue was observed, which indicated a mono-methylated lysine residue. **f** Western blotting for anti-HA immunoprecipitates from HEK293 cells expressing HA-AE-W (lane 2), HA-AEK24R (lane 3), or HA-AEK43R (lane 4). IP Immunoprecipitation, WB Western blotting; AE AML1-ETO. The “Input” of each Fig is the immunoblot analysis for whole cell extracts to show the target protein levels. meK43: our anti-AML1-ETO Lys43 antibody; meK: commercial lysine methylation antibody. The data are representative of 3 independent experiments. Source data are included in the Source Data file

Previous studies have shown that lysine methylation is important for the activities of transcription factors^15^. Given that AML1-ETO is a leukemia-initiating transcription factor containing 31 lysine residues, we explored the possibility of AML1-ETO lysine methylation in leukemia cells. We subjected the anti-ETO immunoprecipitates from Kasumi-1 and SKNO-1 cells (Supplementary Fig. 2a) to Western blotting with a methyl-lysine specific antibody (anti-meK, labeled as meK in Fig. 2), and observed a robust signal on AML1-ETO (Fig. 2a), but not ETO (not shown). However, this signal was largely diminished upon AML1-ETO knockdown (Fig. 2b). The anti-HA pull-down followed by probing with anti-meK revealed a unique AML1-ETO band (Supplementary Fig. 2b) in HEK293 cells expressing **HA-AE-W** (W, wild type). Importantly, ETO pull-down followed by probing with anti-meK in AML-ETO-positive patient cells identified a strong AML1-ETO band (Fig. 2c). These results indicate that AML1-ETO is endogenously methylated in AML cells.

To map the methylated lysine residue(s), we established and transiently expressed a series of His-tagged constructs, including **His-AE-W** (wild type), **His-AE-ΔNHR1** (no residues 264−359), **His-AE-ΔNHR2** (no residues 481–547), and **His-AE-ΔRUNT** (no residues 49−177; Fig. 2d, upper). Immunoprecipitation (IP) by anti-His followed by probing with anti-meK revealed that deletion of the NHR1 (Fig. 2d, lane 2), but not the NHR2 or RUNT (Fig. 2d, lanes 3, 4) domain, impairs AML1-ETO methylation. Furthermore, AML1-ETO9a, a truncated isoform of AML1-ETO lacking the NHR3 and NHR4 domains (HA-AE9a)^29^, was methylated (Supplementary Fig. 2c, lane 2). These data suggest that the NHR1 domain is indispensable for AML1-ETO methylation. Given that AML1-ETO consists of 1−177 residues of AML1 and nearly the full-length ETO, and because deletion of the RUNT domain (residues 49 to 177) or NHR2 domain did not affect AML1-ETO methylation, no methylation on ETO (Supplementary Fig. 4f) supports Lys24 and Lys43 as the only candidate methylation sites. Indeed, mass spectrometry (MS) analysis identified AML1-ETO monomethylation at Lys43 within the AML1 portion in Kasumi-1 cells (Fig. 2e). Consistently, Western blotting detected a strong methylation signal in cells expressing **HA-AE-W** (wild type), however, substitution of Lys43 (**HA-AEK43R**), but not Lys24 (**HA-AEK24R**), with arginine in the full-length AML1-ETO abolished methylation (Fig. 2f). These data indicate that AML-ETO monomethylation occurs exclusively at Lys43. To further validate the AML1-ETO Lys43 methylation and acetylation, we developed specific antibody for methylated AML1-ETO at K43 (anti-methylated K43-AML1-ETO, meK43). Like the results using the commercial methyl-lysine antibody (labeled as meK in Fig. 2), our specific anti-methylated K43-AML1-ETO antibody (labeled as meK43 in Fig. 2) detected a robust signal on AML1-ETO.

### AML1-ETO and EZH1 interact through the NHR1 and WD domains

**Fig. 3:**
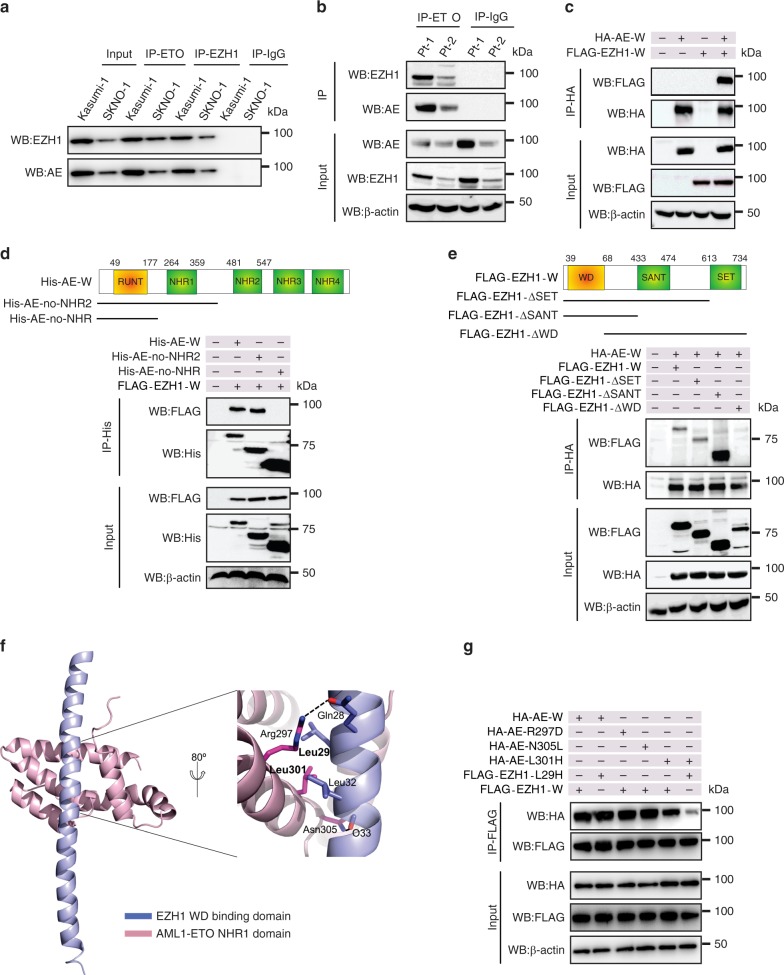
NHR1 domain of AML1-ETO interacts with the WD domain of EZH1. **a** Western blotting for anti-ETO and anti-EZH1 immunoprecipitates (lanes 3–6) from Kasumi-1 and SKNO-1 cells. IgG pull-down performed in parallel (lanes 7, 8) was used as a negative control. **b** Western blotting for anti-ETO immunoprecipitates from AML1-ETO-positive patient primary cells. IgG pull-down performed in parallel was used as a negative control. Pt, patient. **c** HEK293 cells were transfected for 48 h with HA-AE-W (wild type), FLAG-EZH1-W (wild type) alone or both. The anti-HA immunoprecipitates were subjected to Western blotting. **d** Upper: diagram of AML1-ETO domain constructs; lower: Western blotting for anti-His immunoprecipitates from HEK293 cells expressing His-AE-W (wild type), His-AE-no-NHR2, His-AE-no-NHR with FLAG-EZH1-W (wild type). **e** Upper: diagram of EZH1 domain constructs; lower: HEK293 cells were transfected for 48 h with HA-AE-W (wild type) and FLAG-EZH1-W (wild type), FLAG-EZH1-ΔSET, FLAG-EZH1-ΔSANT, or FLAG-EZH1-ΔWD. The anti-HA immunoprecipitates were subjected to Western blotting. **f** A model structure of the AML1-ETO/EZH1 complex. The EZH1 WD interaction domain and AML1-ETO NHR1 domain are shown in blue and magenta, respectively. A magnified view of the binding interface is shown in the inset, in which the potential interacting residues are represented by a ball-and-stick model. Bold letters indicate the critical amino residues for interactions proven by point mutations. **g** HEK293 cells were transfected for 48 h with HA-AE-W (wild type) and FLAG-EZH1-W (wild type) or the indicated mutant constructs. The anti-FLAG immunoprecipitates were subjected to Western blotting. IP Immunoprecipitation, WB Western blotting, AE AML1-ETO; The “Input” of each Fig is the immunoblot analysis for whole cell extracts showing the target protein levels. The data are representative of 3 independent experiments. Source data are included in the Source Data file

To elucidate the mechanisms underlying AML1-ETO Lys43 methylation (methylated K43), we sought to identity a co-factor that physically interacts with AML1-ETO and simultaneously possesses protein lysine methyltransferase (PKMT) activity. We used co-immunoprecipitation to verify the AML1-ETO-EZH1 interaction in Kasumi-1 and SKNO-1 cells (Fig. 3a), AML1-ETO-positive patient cells (Fig. 3b) and HEK293 cells co-expressing **HA-AE-W** (wild type) and **FLAG-EZH1-W** (wild type) (Fig. 3c). As EZH1 was also detected in the ETO immune-complex (Supplementary Fig. 3a), the AML1-ETO-EZH1 interaction might occur within the ETO portion. However, no protein interaction of AML1-ETO with EZH2 could be demonstrated in cells co-expressing **AE-W** (wild type) and **EZH2-W** (wild type). Collectively, these findings support EZH1 as an interacting partner of AML1-ETO in leukemia cells.

To locate the EZH1-binding sites within AML1-ETO, we co-expressed **FLAG-EZH1-W** (wild type) with **His-AE-W** (wild type) or AML1-ETO domain deletion mutants (Fig. 3d, upper). Co-IP verified the binding of full-length AML1-ETO to EZH1 (Fig. 3d, lane 2). Although deletion of NHR2, NHR3, and NHR4 had no effect on EZH1 binding (Fig. 3d, lane 3), NHR1 deletion abrogated this binding (Fig. 3d, lane 4). Further, EZH1 was detected in the AML1-ETO9a complex (Supplementary Fig. 3b, lane 4), like the full-length AML1-ETO (Supplementary Fig. 3b, lane 5). Together, these results indicate that the NHR1 domain is necessary for the AML1-ETO-EZH1 interaction.

To identify EZH1 domains that bind AML1-ETO, we constructed plasmids designed to express different EZH1 portions (Fig. 3e, upper), including **FLAG-EZH1-ΔSET** (no residues 613–734), **FLAG-EZH1-ΔSANT** (no residues 433–734, only WD domain), and **FLAG-EZH1-ΔWD** (no residues 39–68). Co-IP revealed the association between **AML1-ETO** and **EZH1-W** (wild type), **EZH1-ΔSET** or **EZH1-ΔSANT** (Fig. 3e, lanes 2–4), but WD deletion impaired the AML1-ETO-EZH1 complex (Fig. 3e, lane 5). Given that the WD domain alone is co-precipitated with AML1-ETO (Fig. 3e, lane 4), these findings support the idea that the WD domain is required and sufficient for EZH1 to physically interact with the NHR1 domain of AML1-ETO. Notably, as shown in Supplementary Fig. 3c, 12 residues in EZH1 WD domain (residues 45–73) are different from those of EZH2 (residues 39–67), and there is 57.1% (16/28) variation of the WD domain between EZH2 and EZH1. This may partially explain why AML1-ETO and EZH2 do not have physical interactions, which warrants further investigations.

### Complex model structure of the NHR1 and WD domains

To further characterize the AML1-ETO and EZH1 complex, we expressed **GST-EZH1-WD** (WD [EBD] domain only) and **His-AE-NHR1** (NHR1 domain only) in BL21 *E. coli* (Supplementary Fig. 3d, upper). The GST pull-down assays revealed that **GST-EZH1-WD** co-precipitated with **His-AE-NHR1**, but GST alone did not (Supplementary Fig. 3d, lower), suggesting a direct protein interaction of AML1-ETO and EZH1. We modeled the complex structures of EZH1-EBD and AML1-ETO-NHR1 using a protein-docking program^30,31^. The NMR structures of AML1-ETO-NHR1 are known (PDB access codes 2PP4, 2H7B, and 2KNH) and the EZH1-WD interaction domain structure was built (a simple long alpha-helix) using the same region of the EZH2 structure (PDB access code 2QXV) as a template. Protein docking simulation was conducted to model potential interactions. The top solution displayed highly favorable interactions that involved leucine zipper-like hydrophobic interactions made by Leu29 and Leu32 from EZH1 and Leu301 from AML1-ETO, and a set of hydrogen bonds formed by Gln28 and the carbonyl oxygen of Gln33 from EZH1 and Arg297 and Asn305 from AML1-ETO, respectively (Fig. 3f).

To test our model structure, we introduced point mutations at the interacting residues and conducted binding studies along with the wild type proteins. As shown in Fig. 3g, among the single mutations on each protein, such as R297D, L301H, and N205L on AML1-ETO and L29H on EZH1, only the L301H mutant showed a noticeable reduction in protein interaction (lane 5). This indicates a more significant contribution of AML1-ETO leucine zipper-like hydrophobic interactions to overall binding and recognition compared to relatively weaker hydrogen bond interactions. While the mutations involving its interacting partner alone (EZH1-L29H) did not alter the extent of protein interactions (lane 2), a greater reduction was observed (lane 6) when both residues were mutated (AML1-ETO-L301H and EZH1-L29H, Fig. S3e), which indicated additive effects of these mutations. These results support our model structure, although it warrants further confirmation by actual structural determination of the complex.

### EZH1 is an AML1-ETO lysine methyltransferase

**Fig. 4:**
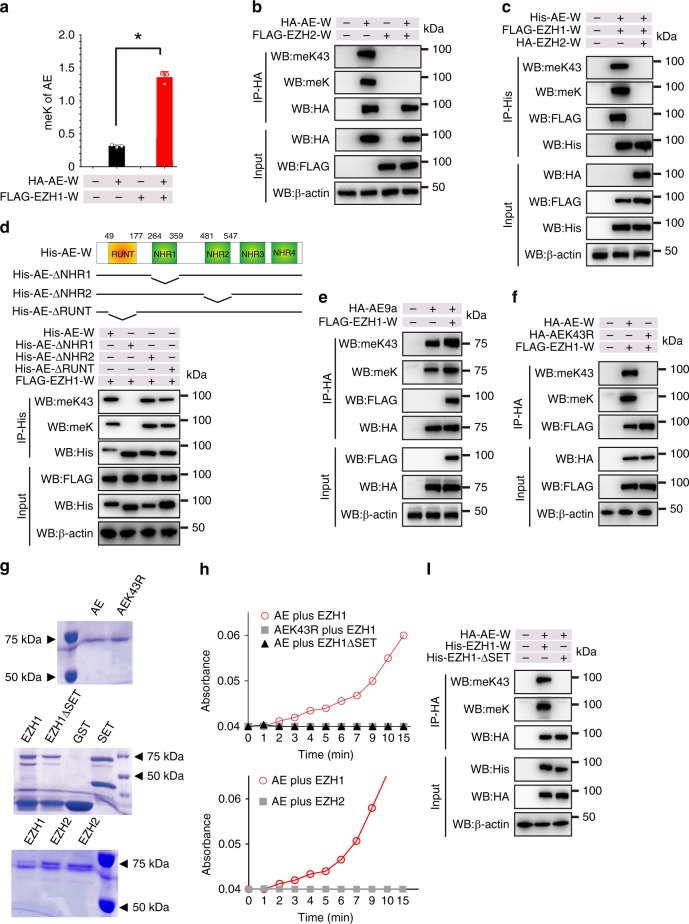
EZH1 mediates AML1-ETO Lys43 methylation in vitro and in vivo. **a** Quantification of AML1-ETO lysine methylation (*n* = 3). HEK293 cells were transfected with HA-AE-W (wild type) alone or plus FLAG-EZH1-W (wild type) for 48 h. The anti-HA immunoprecipitates were subjected to Western blotting. Data are expressed as mean values ± S.D. *P < 0.05. **b** Western blotting for anti-HA immunoprecipitates from HEK293 cells expressing HA-AE-W (wild type), FLAG-EZH2-W (wild type), or both. **c** Western blotting for anti-His immunoprecipitates from HEK293 cells expressing vehicle, His-AE-W (wild type) with FLAG-EZH1-W (wild type), or His-AE-W (wild type) with FLAG-EZH1-W (wild type) plus HA-EZH2-W (wild type). **d** Upper: diagram of AML1-ETO domain constructs; lower: Western blotting for anti-His immunoprecipitates from HEK293 cells expressing His-AE-W (wild type), His-AE-ΔNHR1, His-AE-ΔNHR2, or His-AE-ΔRUNT plus FLAG-EZH1-W (wild type). **e** Western blotting for anti-HA immunoprecipitates from HEK293 cells expressing either HA-AE9a, FLAG-EZH1-W (wild type) alone or both. **f** Western blotting for anti-HA immunoprecipitates from HEK293 cells expressing HA-AE-W (wild type), HA-AEK43R, or plus FLAG-EZH1-W (wild type). **g** Coomassie blue-stained gels showing the proteins used for in vitro methylation assays. **h** In vitro methylation assays. Upper: the purified GST-AE, GST-AE-K43R proteins were incubated with GST-EZH1-W (wild type) or GST-EZH1-ΔSET in the presence of SAM at 37 °C. Lower: the purified GST-AE proteins were incubated with GST-EZH1-W (wild type) or GST-EZH2-W (wild type) in the presence of SAM at 37 °C. The SAM labeled AE was measured by Epoch 2 microplate spectrophotometer. **i** HEK293 cells were transfected with HA-AE-W (wild type) plus His-EZH1-W (wild type) or His-EZH1-ΔSET for 48 h; anti-HA immunoprecipitates were subjected to Western blotting. *P *<* 0.05; Fig. **a**, one-way ANOVA; IP Immunoprecipitation, WB Western blotting, SAM S-adenosyl methionine, AE AML1-ETO. The “Input” of each Fig is the immunoblot analysis for whole cell extracts to show the target protein levels. meK43: our anti-AML1-ETO Lys43 antibody; meK: commercial lysine methylation antibody. The data are representative of 3 independent experiments. Source data are included in the Source Data file

To investigate whether EZH1 is responsible for Lys43 methylation, we co-expressed **HA-AE-W** (wild type) and **FLAG-EZH1-W** (wild type). The results of Western blotting with anti-meK revealed a higher level of methylation compared to that in cells expressing AML1-ETO alone, which might result from endogenous EZH1 (Fig. 4a; Supplementary Fig. 4a). We used three shRNAs targeting *EZH1* at different sites and selected the most efficient shRNA3 (Supplementary Fig. 4b) to assess the impacts of EZH1 knockdown (Supplementary Fig. 4c). We found that EZH1 shRNA, but not scramble, decreases Lys43 methylation in ETO immunoprecipitates (Supplementary Fig. 4d). This was further confirmed by the reduced methylation of AML1-ETO and AML1-ETO9a in HA-pulldown experiments (Supplementary Fig. 4e). Because EZH1 and EZH2 are key components of the PRC2 complex^19^ with overlapping functions^32^, we investigated whether EZH2 also participates in AML1-ETO methylation. Intriguingly, co-IP revealed that EZH2 overexpression impairs AML1-ETO Lys43 methylation (Fig. 4b). Because a physical interaction of EZH2 and AML1-ETO was not detected, EZH2 appeared to indirectly suppress AML1-ETO Lys43 methylation. Indeed, the co-IP results revealed that EZH2 disrupts the AML1-ETO-EZH1 association (Fig. 4c, lane 3). These results support the idea that EZH1 mediates the AML1-ETO Lys43 methylation.

### The SET and NHR1 domains account for AML1-ETO methylation

To identify the AML1-ETO domain(s) involved in Lys43 methylation, we co-expressed EZH1 with **His-AE-W** (wild type), **His-AE-ΔNHR1**, **His-AE-ΔNHR2**, or **His-AE-ΔRUNT** (Fig. 4d, upper). The AML1-ETO pulldown with anti-His revealed a comparable lysine methylation for AML1-ETO full length (Fig. 4d, lane 1) and mutants with deletion of NHR2 or RUNT (Fig. 4d, lanes 3 and 4). However, deletion of the NHR1 domain appeared to abrogate AML1-ETO methylation (Fig. 4d, lane 2), indicating that the NHR1 domain is required for EZH1-induced methylation, which is in line with the finding that the removal of NHR1 disrupted the AML1-ETO-EZH1 interaction. Notably, ETO itself can interact with EZH1, but no methylation was detected in cells co-expressing **FLAG-ETO-W** (wild type) and **His-EZH1-W** (wild type) (Supplementary Fig. 4f). Moreover, AML1-ETO9a exhibited a higher level of lysine methylation when co-expressed with EZH1 (Fig. 4e, lane 3). Collectively, these findings suggest that the site of AML1-ETO lysine methylation is outside ETO, but within the AML1 portion. This conclusion was further substantiated by our data showing that Lys43 substitution with arginine blocks the EZH1-mediated AML1-ETO methylation that could not be rescued by EZH1 overexpression (Fig. 4f, lane 3), although **HA-AE-W** (wild type) was efficiently methylated by **FLAG-EZH1-W** (wild type) (Fig. 4f, lane 2). In addition, when the AML1-ETO and EZH1 interaction was disrupted by the mutations of L301 on AML1-ETO and L29 on EZH1 (see Fig. 3g), a greater reduction of EZH1-mediated AML1-ETO methylation was observed with no obvious changes in AML1-ETO acetylation (Supplementary Fig. 4g, lane 4).

To further understand AML1-ETO methylated K43, we conducted in vitro methylation assays using bacterial expressed GST-tagged full-length EZH1. **GST-AML1-ETO** (GST-AE) was constructed by fusing GST to a region between amino acids 1 and 143 of AML1-ETO. The Coomassie blue-stained gels (Fig. 4g) indicated a comparable amount of the proteins used in the methylation assays. When the AML1-ETO protein was incubated with EZH1ΔSET or full-length EZH1 plus S-adenosyl-L-methionine (SAM), a methylation donor, GST-AE, but not GST-AEK43R, was labeled with SAM in the presence of full length EZH1, whereas no signal was detected for AE plus EZH1ΔSET (Fig. 4h). These findings were in line with the co-IP results showing that deletion of SET domain disrupts the AML1-ETO-EZH1 protein interaction (Fig. 4i, lane 3). When the AML1-ETO protein was incubated with SAM plus either full-length EZH1or full-length EZH2, GST-AE with full-length EZH1, but not EZH2, was labeled with SAM (Fig. 4h, lower panel), which was consistent with the co-IP results (see Fig. 4b). Collectively, these results indicate that EZH1 directly methylates AML1-ETO at Lys43 through the SET domain.

### Dynamic regulation of AML1-ETO Lys43 methylation

Intriguingly, EZH1 binds the NHR1 domain and methylates Lys43, overlapping the sites where p300 physically and functionally interacts^5^. Because the acetylation and methylation on the same residue of histone proteins are coupled to opposing biological outcomes, AML1-ETO lysine acetylation by p300 could potentially interfere with AML1-ETO lysine methylation by EZH1. To test this, **HA-AE-W** (wild type) and **FLAG-p300** were co-expressed in HEK293 cells and the anti-HA immunoprecipitates were subjected to Western blotting. The strong bands detected by anti-FLAG or anti-Ace verified the AML1-ETO interaction with p300 and AML1-ETO Lys43 acetylation (Supplementary Fig. 4h), which might have resulted from the decreased EZH1 binding to AML1-ETO (not shown). Importantly, ectopic p300 expression diminished Lys43 methylation, whereas EZH1 overexpression induced a noticeable reduction of Lys43 acetylation (Supplementary Fig. 4i, lane 3). Therefore, we propose that EZH1 and p300 competitively bind AML1-ETO and modify the Lys43 residue.

Because EZH1, EED, and SUZ12 constitute the core of PRC2^33^, we examined whether the EZH1 and AML1-ETO protein interaction occurs in a PRC2-dependent manner. Co-IP in Kasumi-1 and SKNO-1 cells revealed the presence of EED and SUZ12 in the EZH1 complex; however, anti-ETO failed to pull down EED or SUZ12 (Supplementary Fig. 4j). When EED and SUZ12 were knocked down, no obvious changes in methylated K43 were observed (Supplementary Fig. 4k). These results support the idea that EZH1-dependent K43 methylation possibly occurs in a PRC2 complex-independent manner. Given that EZH2 overexpression diminishes AML1-ETO K43 methylation (see Fig. 4b), these results suggest a dynamic feature of AML1-ETO K43 methylation, which certainly merits further investigation.

### K43 methylation fails to alter AML1-ETO protein interactions

Because AML1-ETO activities greatly depend on its dynamic interactions with various factors, we next sought to investigate whether EZH1 impacts the AML1-ETO interaction with other proteins (e.g., DNMTs and HDACs). When EZH1 and AML1-ETO were co-expressed, more endogenous DNMT3a and HDAC1 were pulled down with AML1-ETO compared to that by AML1-ETO only (Supplementary Fig. 4l, m), which hints to an enhanced AML1-ETO binding affinity. To determine whether methylated K43 changes the protein-interacting property of AML1-ETO, we expressed AE and AEK43R in HEK293 cells. Co-IP revealed that AE and AEK43R are pulled down together with EZH1, p300, and HDAC1 at similar levels with a mild increase of DNMT3a (Supplementary Fig. 4n). EZH1 overexpression did not obviously change the affinity of binding with interacting partners for HA-AEK43R compared to HA-AE-W (wild type) (Supplementary Fig. 4o).

### Lys43 methylation enhances AML1-ETO repressive activity

**Fig. 5:**
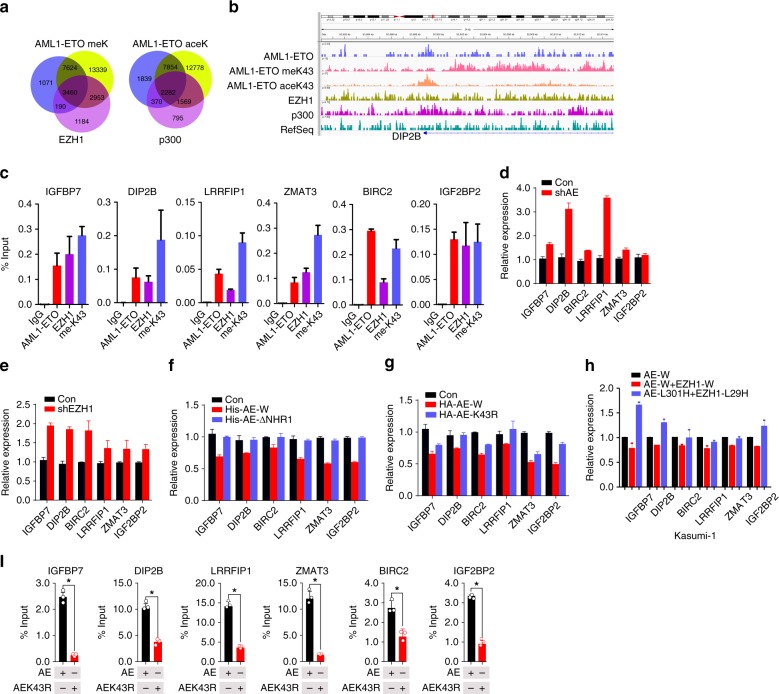
AML1-ETO Lys43 methylation is essential for AML1-ETO transcriptional activity. **a** Venn diagram illustrating the number of genes in which the peaks of AML1-ETO co-localize with those of EZH1, p300, meK43, or aceK43 in Kasumi-1 cells determined by anti-AML1-ETO (specific to AE, but not wild-type ETO or AML1), anti-EZH1, anti-p300, anti-meK43 (methylated AEK43, our developed antibody) or anti-aceK43 (acetylated AEK43, our developed antibody). **b** UCSC Genome Browser image depicting the human methylated K43-AML1-ETO and EZH1 loci demonstrating a co-localization of peaks at DIP2B promoter. **c** ChIP-qPCR showing the co-occupancy of AML1-ETO, EZH1, me-K43 at AML1-ETO target promoters in Kasumi-1 cells using an AML1-ETO specific antibody, anti-EZH1 or anti-me-K43 (*n* = 3). **d**, **e** qPCR for changes in the AML1-ETO and EZH1 target gene expression in Kasumi-1 cells with knockdown of AE (**d**) or EZH1 (**e**) (*n* = 3). **f**, **g** qPCR for changes in AML1-ETO and EZH1 target gene expression in HEK293 cells transfected with AE-W (wild type), AE-ΔNHR1 or AEK43R (*n* = 3). **h** qPCR for changes in AML1-ETO and EZH1 target gene expression in Kasumi-1 cells transfected with the indicated mutant constructs (*n* = 3). **i** ChIP-qPCR assessing the enrichment of AE and AEK43R in the target promoters (*n* = 3). *P *<* 0.05; Fig. **i**, one-way ANOVA; AE AML1-ETO, meK43 methylated AEK43, aceK43 acetylated AEK43. The data are representative of 3 independent experiments. Data are expressed as mean values ± S.D. Source data are included in the Source Data file

To identify global targets with occupancy of AML1-ETO, EZH1, p300, methylated-AEK43, and acetylated-AEK43, we conducted chromatin IP (ChIP) assays using our antibodies specific for the acetylated and mono-methylated AML1-ETO Lys43 (anti-acetylated K43-AML1 and anti-methylated K43-AML1-ETO) in Kasumi-1 cells followed by massive parallel DNA sequencing (ChIP-seq). The ChIP-seq data revealed that AML1-ETO occupies 3460 genes with EZH1 and methylated-AEK43, and 2282 genes with p300 and acetylated-AEK43 (Fig. 5a). As a readout, we showed that both methylated K43-AML1-ETO and EZH1 are enriched in *DIP2B* promoter (Fig. 5b). Using DAVID pathway analysis, the co-targets of AML1-ETO and EZH1 were classified into various cellular signaling pathways that are involved in cell cycle, proliferation and migration (Supplementary Fig. 5a). The genes in these pathways included *IGFBP7*, *DIP2B*, *BIRC2*, *LRRFIP1*, Z*MAT3*, and *IGFBP2*, which are tumor suppressor genes, and some are known to be transcriptionally repressed by AML1-ETO^5^. ChIP-qPCR in Kasumi-1 cells showed a strong correlation among AML1-ETO, EZH1, and methylated-AEK43 enrichments in these genes (Fig. 5c, Supplementary Fig. 5b), thus validating the ChIP-Seq results. These findings suggest that EZH1 and AML1-ETO co-occupy the promoters of transcriptionally repressed genes.

To study the impacts of methylated K43 on global gene expression, and to correlate these changes with AML1-ETO targets identified by ChIP-Seq, we generated gene expression profiles by RNA-seq in AML1-ETO-depleted Kasumi-1 cells. We overlapped the differentially expressed genes (1.5-fold threshold, *P* *<* 0.05 by analysis of Variance) with binding sites for AML1-ETO and methylated K43 or acetylated K43-AML1-ETO. These experiments identified 200 activated and 170 repressed genes (with AML1-ETO binding sites, Supplementary Data 1 and 2). Importantly, methylated K43-AML1-ETO bound the majority of the 170 repressed genes (137 genes, 80.6%; Supplementary Data 3), whereas acetylated K43-AML1-ETO was associated with the majority of the 200 activated genes (168 genes, 84%; Supplementary Data 4). These findings support the idea that AML1-ETO methylation mainly acts as a gene repressor, but its acetylation is a gene activator^5^.

Because AML1-ETO is generally thought to be a gene repressor^5,34^, and we found that methylated K43-Methylated-AML1-ETO is enriched in the promoters of AML1-ETO-suppressed genes, next we sought to determine how Lys43 methylation alters AML1-ETO-dependent repressive activities. First, the results of qPCR revealed a substantial increase in methylated K43 and EZH1 co-targets, including *IGFBP7*, *DIP2B*, *BIRC2*, *LRRFIP1*, *ZMAT3*, and *IGF2BP2*, in SKNO-1 cells with AML1-ETO or EZH1 knockdown (Fig. 5d and e). Second, because NHR1 deletion or Lys43 mutation disrupted methylated K43, we expressed **AE-W** (wild type), **AE-ΔNHR1**, or **AEK43R** in HEK293 cells. Although **AE-W** (wild type) obviously repressed the above genes, **AEK43R** and **AE-ΔNHR1** countered this repressive activity (Fig. 5f, g). These findings were further verified in Kasumi-1, SKNO-1, and SKNO-1 siAE cells (Supplementary Fig. 5c–g). Importantly, the introduction of EZH1 into AEK43R failed to rescue AML1-ETO repressive effects (Supplementary Fig. 5i). Moreover, point mutations on interacting residues (L301H and L29H) also impaired AML1-ETO-dependent gene repression (Fig. 5h; Supplementary Fig. 5h). Mechanistically, methylated K43 loss by mutations (AEK43R, L301H, and L29H) markedly inhibited AML1-ETO binding to these genes’ promoters (Fig. 5i, Supplementary Fig. 5j–l). Given that Lys43 mutation did not largely affect the AML1-ETO interaction with its co-partners, these findings indicate that AML1-ETO-dependent transcription relies more on Lys43 methylation than protein interactions.

### Lys43 methylation is essential for AML cell proliferation

**Fig. 6:**
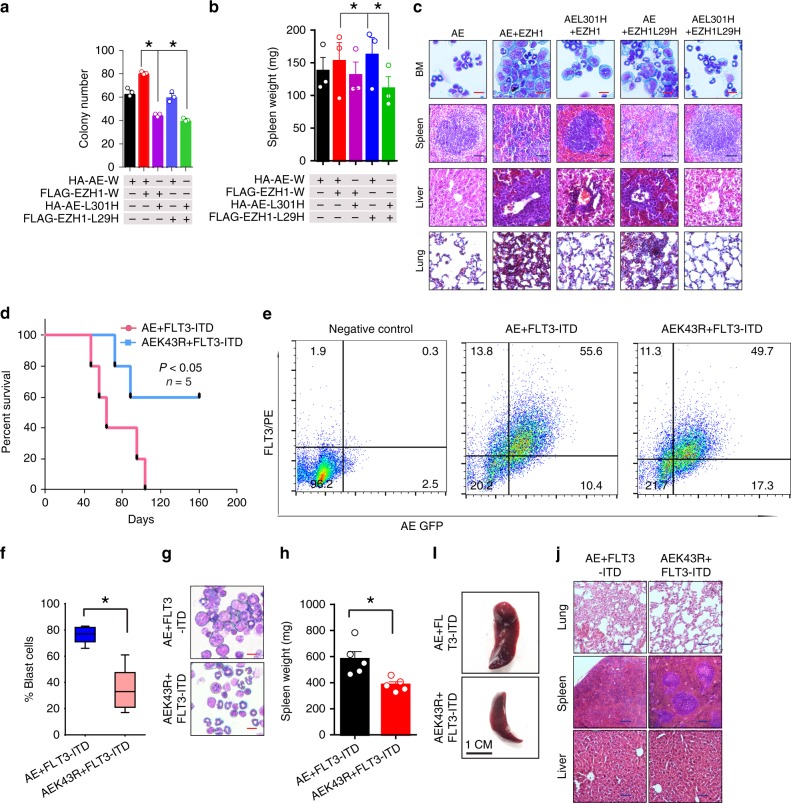
EZH1-mediated Lys43 methylation is essential for leukemic cell expansion. **a** Colony-forming assays in C1498 cells transfected with HA-AE-W (wild type) and FLAG-EZH1-W or the indicated mutant constructs (*n* = 3). **b** Graph illustrates the average of the spleen weights from leukemic mice (*n* = 3 mice/group) engrafted with C1498-transfected cells (0.1 × 10^6^, from **a**). **c** Wright/Giemsa staining of BM and H&E staining of sections from the spleens, lungs, and livers of leukemic mice engrafted with the transfected C1498 cells (from **a**). Scale bars represent 50 µm (red) and 100 µm (blue). **d** Kaplan–Meier survival curves of the engrafted mice. The mice transplanted with bone marrow cells co-expressing AML1-ETO plus FLT3-ITD developed fatal leukemia (*n* = 5). **e** BM cells transduced with AML1-ETO plus FLT3-ITD or AML1-ETOK43R plus FLT3-ITD viruses were injected into C57BL/6 J mice (*n* = 5 mice/group) through the tail vein. FACS analysis of the engrafted recipient BM cells from the representative disease mice. **f**, **g** Blast cell percentage by immunophenotype analysis (**f**) and morphological analysis (**g**) of hematopoietic cells in bone marrow from the representative disease mice (from **d** and **e**). Scale bars represent 50 µm (red). **h** Graph illustrates the average of the spleen weights (from **d** and **e**) (*n* = 5). **i** Representative external views of the spleens from the leukemic mice (from **d** and **e**). **j** H&E staining of sections from the spleens, lungs, and livers of the leukemic mice (from **d** and **e**). Note, Fig. **a**, data are expressed as mean values ± S.E.M. of triplicate samples from three independent experiments; Fig. **b**, **h**, graphs are the average of spleen weight and data are expressed as mean values ± S.D; Fig. **j**, original magnification ×200 and scale bars represent 100 µm (blue); BM bone marrow, AE AML1-ETO; *P *<* 0.05; Fig. **a**, **b**, **f**, **h**, one-way ANOVA; **d**, Log-rank test; For box-whisker plots, box edges correspond to 25th and 75th percentiles, lines inside the box correspond to 50th percentiles, and whiskers include extreme data points. Source data are provided as a Source Data file

Because K43 is subjected to acetylation by p300 and methylation by EZH1, to determine whether methylated K43 influences AML1-ETO-related cell growth, as a proof of concept, we expressed **AEL301H** and **EZH1L29H** in C1498 cells (Supplementary Fig. 6a). While cells expressing a single mutation showed a moderate reduction in cell colony formation, those expressing both AEL301H and EZH1L29H had the lowest colony number (Fig. 6a). Mice engrafted with cells expressing AEL301H plus EZH1L29H manifested less aggressive illness compared to that observed in mice injected with cells transfected with AML1-ETO plus EZH1. This was reflected in the smallest spleens (Fig. 6b) and the most moderate infiltration of leukemic cells into the bone marrow, lungs, spleens, and livers of the recipients (Fig. 6c and S6b).

To further assess the contribution of Lys43 methylation to AML1-ETO leukemogenesis, we conducted bone marrow (BM) transplantation studies using BM cells from C57BL/6 mice (*n* = 5 mice/group) transduced with **AML1-ETO** plus **FLT3-ITD** or **AML1-ETOK43R** plus **FLT3-ITD** virus. Expression of the AML1-ETO and FLT3-ITD was detected using qPCR in BM cells before injection (Fig. S6c). Then the C57BL/6 mice (*n* = 5 mice/group) were lethally irradiated with 9 Gy, given a split dose separated by 4 h, and were injected through the tail vein with a mixture of AML1-ETO+/FLT3-ITD+ cells or AML1-ETOK43R+/FLT3-ITD+ (range 3 × 10^5^ to 7.5 × 10^5^ cells) with nontransduced BM cells (range 2.3 × 10^5^ to 1.1 × 10^6^). All mice receiving AML1-ETO+/FLT3-ITD+ transduced BM cells had a worse prognosis with a median latency time of 86 days post-transplantation (Fig. 6d). The white blood cell (WBC) counts were highly elevated in AML1-ETO+/FLT3-ITD+ mice compared with AML1-ETOK43R+/FLT3-ITD+ mice (Supplementary Fig. 6d), and the bone marrow had a high proportion of blasts (Fig. 6e–g). Consequently, the mice engrafted with BM cells expressing AML1-ETO plus FLT3-ITD were moribund, cachectic, and suffered from splenomegaly (median spleen weight 581 mg) with infiltration of leukemic blasts that were readily detectable in bone marrow and other organs (Fig. 6h–j). Collectively, these findings support the idea that Lys43 methylation partially regulates AML1-ETO leukemogenicity.

## Discussion

In this study, we discovered lysine methylation as a previously unknown post-translational modification of the chimeric transcription factor AML1-ETO. We identified EZH1 as a AML1-ETO interacting partner and demonstrated the non-histone PKMT activity of EZH1 in leukemia. We showed that the NHR1 domain of AML1-ETO directly interacts with the WD domain of EZH1, and that this protein interaction is required for EZH1 to methylate AML1-ETO Lys43. While EZH1 recruitment enhanced the capability of AML1-ETO to interact with co-regulators, methylated K43 appeared to be more essential for the efficacy of AML1-ETO-induced transcriptional repression in leukemia cells.

The functions of AML1-ETO critically depend on its capability to interact with additional factors coupled by posttranslational modifications. Among these interacting factors, p300 and PRMT1 mediate AML1-ETO lysine acetylation or arginine methylation, respectively, in turn promoting AML1-ETO functions^2,5^. These findings indicate that AML1-ETO-dependent transcription is a dynamic process that is fine-tuned by protein interactions and/or modifications. This idea is substantiated by our discoveries showing that (1) EZH1 directly interacts with AML1-ETO; (2) analogous to p300 and PRMT1, EZH1 directly methylates AML1-ETO at Lys43; and (3) EZH1-dependent methylated K43 augments the AML1-ETO-induced repression. Intriguingly, p300 also binds and modifies the same site^5^ as does EZH1 on the AML1-ETO protein. The findings that p300 overexpression diminishes EZH1 binding to AML1-ETO, and that enforced p300 expression decreases methylated K43, while EZH1 overexpression inhibits acetylated K43, reflect an antagonistic effect of meK43 and acetylated K43 on AML1-ETO function (Supplementary Fig. 7). This is consistent with the documented competitiveness of acetylation and methylation on the same residue of histones^35^. Notably, although both are crucial for AML1-ETO function, we demonstrate that the loss of AML1-ETO methylated K43 does not significantly interfere with the AML1-ETO protein interaction, but markedly diminishes AML1-ETO activities, and that the blockage of AML1-ETO-induced transcription by loss of AML1-ETO methylated K43 could not be rescued by the introduction of EZH1, in which the interacting properties and acetylated K43 of AML1-ETO are retained. These findings strengthen the idea that Lys43 methylation plays a more seminal role than the protein interactions in determining AML1-ETO-dependent transcriptional properties. This is consistent with a previous report showing that the acetylation of AML1-ETO itself, but not p300, is the key step in AML1-ETO-induced leukemogenesis^5^.

Recently, lysine methylation of non-histone proteins (e.g., p53, TAF10, GATA4, RelA) by HMKTs has garnered more attention due to its importance in human tumorigenesis^15^. However, whether and how lysine amino acids of fusion proteins are methylated and the biological outcomes of their lysine methylation have not been investigated. Our study demonstrates that AML1-ETO exclusively displays Lys43 monomethylation, which crucially regulates AML1-ETO-dependent gene repression. The uniqueness of AML1-ETO methylated K43 stems from its catalytic enzyme, EZH1. Although multiple HMKTs, SET7/8 SETD6, G9a, and even EZH2, have been identified to methylate non-histone proteins^10,11,13,17^, knowledge regarding EZH1 is still lacking. We were motivated to examine the non-histone PKMT activity of EZH1 in AML1-ETO leukemia, because: (1) EZH1 appears in the AML1-ETO complex; (2) EZH1 and AML1-ETO have a direct protein–protein interaction; (3) EZH1 has a SET domain carrying PMKT activity^26^; (4) EZH1 displays contrasting activity against EZH2 and possesses weaker HMKT activity compared to that of EZH2^25^; and (5) EZH1 appears to have PRC2 complex-independent functions. Our findings strongly support the idea of EZH1 as another non-histone PKMT that is responsible for AML1-ETO methylated K43. This is consistent with the results of in vitro methylation assays and gain-of-function and loss-of-function studies showing that EZH1 activation enhanced, whereas EZH1 dysfunction by gene knockdown, domain deletion or disruption of its interaction with AML1-ETO reduces, Lys43 methylation. Not only did our study reveal methylated K43 as a AML1-ETO modification, but also it increases our understanding of the molecular biology of AML1-ETO leukemogenicity. Further, it reveals another facet of EZH1 that is independent of histone methylation, thus adding a member to the family of histone PKMTs that exhibit methyltransferase activity against non-histone proteins. Due to the difficulty in exploring oncofusion proteins as druggable targets, our findings serve to provide an innovative therapeutic strategy of targeting the non-histone function of EZH1 and lysine methylation for treatment of AML1-ETO leukemia.

In summary, EZH1-mediated Lys43 methylation appears to be essential for AML1-ETO transcriptional regulation. The SET domain offers an enzymatic platform to accomplish Lys43 methylation, and the NHR1 and WD domains provide docking sites for AML1-ETO and EZH1, allowing them to co-localize at the regulatory regions of many target genes, including those involved in cell survival and proliferation. Thus, an accurate balance of the AML1-ETO, p300, and EZH1 ensures the achievement of leukemia initiation, promotion and progression in hematopoietic cells (Supplementary Fig. S7). Therefore, investigation of how the multiple interacting partners, such as EZH2, EZH1, and p300, are orchestrated in determining protein interactions and modifications of AML1-ETO during leukemic transformation and progression remains an exciting challenge for the future.

## Methods

### Plasmids and constructs

**His-AE-W (wild type)**, **His-AE-ΔNHR1**, **His-AE-ΔNHR2**, and **His-AE-ΔRUNT** constructs were obtained by cloning the PCR fragments into the pcDNA4.0 vector. The **His-AE-ΔNHR1** construct lacking residues 264–359 (NHR1 domain deletion, other domains reconnected), the **His-AE-ΔNHR2** construct lacking residues 481–547 (NHR2 domain deletion, other domains reconnected) and the **His-AE-ΔRUNT** construct lacking residues 49–177 were used. Also, **His-AE-no-NHR** (with RUNT domain, without all NHR domains) and **His-AE-no-NHR2** (with RUNT and NHR1 domains) constructs were used in the co-immunoprecipitation (Co-IP) assays. Constructs of the AML1-ETO containing the K43R and K24R mutations were obtained by replacing the lysine residues with arginine using a PCR primer-directed mutagenesis Kit (Agilent Technologies, Inc., Santa Clara, CA) and subcloned into the pUHD vector or pMSCV vector (GFP). The **FLAG-EZH1-ΔSET** construct lacking residues 613–747, the **FLAG-EZH1-ΔSANT** construct lacking residues 433–747 and the **FLAG-EZH1-ΔWD** construct lacking residues 1–68 were obtained by cloning the PCR fragments into the pCMV6 vector. For construction of **His-EZH1-W** (wild type) and **His-EZH1-ΔSET**, the DNA fragments corresponding to wild-type EZH1 and residues 1–612, respectively, were amplified by PCR and subcloned into the pcDNA4.0 vector. **GST-AE-W** (1–443) and **GST-AE-K43R**-(1–443) were obtained by cloning the PCR fragments corresponding to residues 1–443 of AML1-ETO, and AE-K43R into the pGEX-5 × −1 vector, respectively. For construction of **GST-EZH1-ΔSET** (EZH1 without SET domain) and **GST-EZH1-W** (full length), the DNA fragments corresponding to residues 577–728, residues 1–497 and residues 1–734, respectively, were amplified by PCR and subcloned into the pGEX-5 × −1 vector. For construction of **His-AE-NHR1** and **GST-AE-NHR1**, the NHR1 fragment for residues 265–373 of *AML1-ETO* was generated by PCR. After digestion with BamHI and XhoI, the fragment was cloned into the pET41aHT and pGEX-GST vectors. **His-EZH1-WD** and **GST-EZH1-WD**, containing the WD fragment for residues 8–66 of EZH1, were also constructed. All constructs were verified by DNA sequencing. Primer sequences are listed in the Supplementary Table 4. The following expression plasmids were purchased from Addgene (Cambridge, USA): **pCMV-AML1-ETO** (ID 12428), **HA-AE-W** (wild type) (ID 12430), **pSMP-EZH1_2** (ID 36360), **pSMP-EZH1_1** (ID 36359), **pSMP-EZH1_3** (ID 36361), **HA-AE9a** (ID 12433), **pCMV5-AML1B** (ID 12426), **FLAG-ETO-W** (wild type) (ID 12507), **pMSCV-FLT3-ITD** (ID 74499); from OriGene (Rockville, USA): **FLAG-EZH1-W** (wild type) (Myc-DDK-tagged)-Human enhancer of zeste homolog 1 (Drosophila; EZH1; ID RC202367)^36^; The scrambled and shRNA vectors were from BMGC RNAi: **V2LMM_102131-ep300**, **V3LHS_331296-ep300**, **V3LHS_331297-ep300**, **V2LHS_151492-EZH1**, **V2LHS_151493-EZH1**, **V2LHS_151495-EZH1**, **TRCN0000002439**^**−**^**EZH1**, **TRCN000000 2440-EZH1**, **TRCN0000002441-EZH1**, **V2LHS_256397-SUZ12**, **V2LHS_74301-SUZ12**, **V2LHS_23172-EED**, **V2LHS_23174-EED**. The AML1-ETO siRNAs were specific to the conjunction of *AML1/ETO*, but not *AML1* or *ETO* alone:

Sense: 5CCUCGAAAUCGUmACUmGAGUAG3,

Antisense: 5UCUCmAGUmACGAUUUCGAGGUU3.

### Transfections

The HEK293 cells (ATCC, 2 × 10^5^) were seeded into 6-well plates and incubated overnight and then transfected with ~2.5 μg expression, shRNA or the corresponding vehicle vectors using Lipofectamine™ 2000 reagent (Life Technologies, Carlsbad, CA). For gene knockdown in C1498, Kasumi-1, and SKNO-1 cells, electroporation (from ATCC) was employed. Briefly, the cells (1 × 10^7^) were resuspended in 380 μL serum-free and antibiotic-free medium, mixed with 15 μg expression, shRNA and the relevant vehicle vectors in a 0.4 cm gap sterile electroporation cuvette and electroporated using the Gene Pulser Xcell electroporation system (Bio-Rad, Hercules, CA). For co-transfection experiments, the total amount of the transfected DNA was reduced to half of that in the individual agent group, and kept consistent with the co-transfection group by adding vehicle vectors. The siRNAs or respective negative controls (100 nM) were transfected into cells using Lipofectamine™ RNAiMAX Reagent (Life Technologies, Grand Island, NY) according to the manufacturer’s instruction.

### Protein purification and mass spectrometry

Endogenous AML1-ETO from Kasumi-1 cells was immunoprecipitated by anti-ETO, and the eluted proteins were separated by sodium dodecyl sulfate polyacrylamide gel electrophoresis (SDS–PAGE). The protein lysis buffer contains 20 mM Tris-HCL, pH7.5, 1% Triton, 300 mM NaCL, 20% glycerol, 1 mM EDTA and 1 mM EGTA, 0.5 mM DTT, 2.5 mM sodium pyrophosphate, 1 mM β-glycerophosphate, 1 mM Na_3_VO_4_, 50 mM NaF, 1 mM PMSF, and protease inhibitors (protease inhibitor cocktail set III; Calbiochem-Novabiochem, San Diego, CA); wash buffer contains 20 mM Hepes pH7.9, 1 mM EDTA, 20% glycerol, 300 mM NaCL, 0.5% NP40, and 1 mM PMSF. The visible AML1-ETO protein was trypsin digested, separated on an RP C18 column and detected on an Orbitrap Elite LC-MS/MS system. The collected data were processed and searched with Xcalibur (v.2.2) and MASCOT search engine (v.2.5), respectively. Methylation site scoring was carried out using with MASCOT delta and methylation RS algorithms. Peptides identification criteria are set at MASCOT Expectation level of 0.05 (e.g., 95% confidence) for all peptides (http://www.matrixscience.com/help/scoring_help.html). MASCOT Search Engine (Matrix Science, v. 2.4), protein database: SWISSPROT_2014_August. With a minimum of 2 or more peptides to be present for protein ID presense. PTM localization is from peptides with at least 0.05 on the Mascot Expectation scale (Details in Supplementary Methods and Parameters for Proteomic Analysis). Identified PTM peptides were then manually validated by the MS/MS fragmentation patters and location of fragments. The variable modifications were Oxidation (M), propionamide (C), Methyl (K), Dimethyl (K), Trimethyl (K). The peptide mass tolerance was 20 ppm and the fragment mass tolerance was 0.5 Da. Labels corresponding to the b-fragment and y-fragment ions of the peptides were manually assigned. The peptide was ID’ed with Methionine and Lysine as being oxidized and methylated, respectively.

### Co-immunoprecipitation (Co-IP) and Western blot analysis

After various treatments, the whole cellular lysates were prepared by harvesting the cells in 1× cell lysis buffer (20 mM HEPES [pH 7.6], 150 mM NaCl, and 0.1% NP40 supplemented with 1 mM β-glycerophosphate, 1 mM Na_3_VO_4_, 1 mM PMSF, 1 mM NaF, 1 mM benzimedin, and protease inhibitors (protease inhibitor cocktail set III; Calbiochem-Novabiochem, San Diego, CA)^37–39^. Approximately 200 μg nuclear extract or 1 mg total protein lysate was precleared with 70 μL of slurry of protein A or G Dynabeads (Upstate Biotechnology) for 2 h at 4 °C. Dynabeads (70 μL) were coated with 2 to 5 μg antibodies at 4 °C overnight. The wash buffer (1× TBS, 0.1% Tween® 20) was used to elute proteins. The immunoprecipitates and whole cell lysates were subjected to Western blotting using established methods^3,37^. The Western blot was quantified using the Image J Software from the U.S. National Institutes of Health (NIH).

### Glutathione S-transferase (GST) pulldown and gel filtration

The GST-tagged proteins were expressed in *Escherichia coli* BL21 (DE3), and purified with Glutathione Sepharose 4B (GE Healthcare Jupiter, FL), according to the manufacturer’s protocol. The antigen-eluted complex was subjected to gel filtration with a Precision Column PC 3.2/30 that was pre-packed with Superose 6 (GE Healthcare).

### In vitro lysine methylation assays

The GST-tagged AML1-ETO and EZH1 proteins (GST-AE-W, GST-AE-K43R, GST-EZH1-ΔSET and GST-EZH1-WD) were purified with GST Bulk Kit (GE Healthcare), and the methylation assays were conducted using established methods^17^. Briefly, the recombinant proteins of AML1-ETO (GST-AE-W, wild type) or AML1-ETO mutant (GST-AE-K43R) and GST-EZH1 or GST-EZH1-ΔSET were incubated at 37 °C in 0.1 mM S-adenosyl-methionine (AdoMet, Cayman) in methylation buffer using SAM510™ SAM Methyltransferase Assays (G-Biosciences, St. Louis, MO). Reactions were performed at 37 °C and the values were determined using the Epoch 2 Microplate Spectrophotometer (BioTek Instruments, Inc., Winooski, VT).

### Complex model structure of EZH1-EBD and AML-ETO-NHR1

For protein docking studies, three-dimensional (3-D) structures of individual proteins were prepared. The nuclear magnetic resonance (NMR) structures of AML1-ETOL-NHR1 are known (PDB access codes 2PP4, 2H7B, and 2KNH) and the EZH1-WD interaction domain was built by comparative protein structure modeling (a simple long alpha-helix) from the same region of EZH2 structure (PDB access code 2QXV) as template using the program MODELLER^30^. The complex structure was modeled using the GRAMM-X protein docking simulation software^31^. This model structure was derived by smoothing potentials on a fine grid during the global search, followed by the refinement optimization in continuous coordinates, and rescoring with several knowledge-based potential terms.

### Development and characterization of K43 specific antibodies

The polyclonal antibody specific for the acetylated and mono-methylated AML1 at K43 (anti-acetylated K43-AML1 and anti-m K43-AML1) was produced in collaboration with PTM Biolab, Inc. Rabbits were immunized with the acetylated or methylated human AML1 peptide (39-ALAGacKLRSG-47 and 39-ALAGmeKLRSG-47) where aceK43 and meK43 represents the acetylated and methylated K43. Antisera from the immunized rabbits were first depleted with the unmodified peptide (39-ALAGKLRSG-47) and then affinity-purified using the acetylated or methylated peptide.

### ChIP and ChIP-seq

Chromatin immunoprecipitation (ChIP) was performed in Kasumi-1 or in HEK293 cells transfected with different HA-tagged AML1-ETO mutants. The antibodies used were EZH1 and ETO (Santa Cruz Biotechnology), HA-tag and AML1 (Cell Signaling Technology) and are listed in Supplementary Table 5. ChIP sequencing (ChIP-Seq) was performed using established methods^40^. Briefly, ChIP-enriched DNA fragments (anti-AML1, anti-ETO, anti-AML1-ETO specific antibody, anti-EZH1, anti-p300, anti-acetylated AML1-ETO K43, and anti-methylated AML1-ETO K43) were size-selected on an agarose gel. Linkers were then added and the library was amplified using PCR. Each library was loaded onto an individual lane of a Hiseq 2500 next-generation sequencing platform (Illumina). All samples were processed using the standard 50 bp single-end protocol. MACS version 1.4.0alpha2 was used with the default parameters and the appropriate input as control to identify regions of the genome that were significantly enriched for the relevant protein complex. A gene was indicated as bound if one of the MACS identified peaks overlapped an UCSC gene. The specific enrichment was analyzed by qPCR and percent input calculation. We sequenced the total genomic DNA to obtain a reference input profile and used the top 10,000 peaks of *AML1-ETO, EZH1, p300*, methylated-AEK43, or acetylated-AEK43 for comparisons. We determined the number and distribution of peaks relative to the transcription start site of genes that have *AML1-ETO, EZH1*, and methylated-AEK43 binding sites, or *AML1-ETO, p300*, and acetylated-AEK43 binding sites. The primers specific for target gene promoters are listed in Supplementary Table 4.

### RNA isolation and quantitative PCR (qPCR)

Total RNA was isolated using miRNeasy Mini Kit (Qiagen, Valencia, CA). The first strand cDNA synthesis was conducted using the SuperScript® III First-Strand Synthesis System (Invitrogen). AML1-ETO expression was detected using the TaqMan® Gene Expression Assay (Applied Biosystems, Foster City, CA). The expression of EZH1 and other genes was measured using Power SYBR® Green PCR Master Mix (Applied Biosystems). Expression of all genes was determined by the comparative Ct method^41^ using ABL1 or GAPDH levels for normalization. The primers are listed in Supplementary Table 4.

### RNA-Seq library construction and sequencing

Total RNA was obtained from Kasumi-1 cells transfected with *EZH1* shRNA, *AML1-ETO* shRNA, or vehicle vectors. Total RNA (2.0 μg) from each sample was used to construct RNA-Seq libraries using the TruSeq mRNA Sample Preparation Kit (Illumina, San Diego, CA) according to the manufacturer’s instructions. Transcriptome deep sequencing was performed by the Annoroad Gene Technology. RNA-Seq reads were aligned to the GRCh38/hg38 using Tophat 2.0.12 with default parameters. HTSeq v0.6.0 was used to count the number of reads that were mapped to each gene. The expression level of mRNAs in each library was obtained by normalizing reads number to FPKM. DEseq was used to identify significant DE mRNAs, and the significance was declared at fold change > 1.5 and *P* < 0.05.

### Hematoxylin & eosin (H&E) and immunohistochemistry staining

Tumors and tissues were fixed in 10% neutral-buffered formalin, deparaffinized, hydrated, and stained with H&E (Thermo-Scientific) staining with the primary antibodies listed in Supplementary Table 5. A horseradish peroxidase-conjugated goat anti-rabbit antibody was used as the secondary antibody. After developing with 3, 3′-diaminobenzidine, the sections were counterstained with hematoxylin. All sections were observed by microscope and the immunohistochemical signal was quantified using the Image-Pro Plus software (v.4) program (Media Cybernetics).

### Cell cycle assays

The treated cells were stained by the Annexin V (FITC)/7AAD Kit (BD Biosciences, Franklin Lakes, NJ) or propidium iodide and subjected to flow cytometry. For analysis of cell cycle, the cells were washed with PBS and resuspended in 0.4 mL of phosphate-buffered saline (PBS). Then 1 mL of ice-cold absolute ethanol was added. These cells were fixed at –20 °C for a minimum of 2 h, washed with PBS and incubated with propidium iodide (PI, 20 µg mL^−1^) and RNase A (200 µg mL^−1^) for 30 min at room temperature in the dark. These PI-stained cells were analyzed on a Becton Dickinson FACSCalibur flow cytometer (BD Biosciences, San Jose, CA). The intact cells were gated in the FSC/SSC plot to exclude small debris, and cell cycle was determined using ModFit LT software version 4.1.7 (Verity Software House, Inc., Topsham, ME).

### Analysis of gene expression omnibus (GEO) data

Gene expression microarray data from the large cohorts of patients with AML were downloaded from GSE6891^28^, and analyzed for the expression of EZH1. The subjects included are acute myeloid leukemia patients and gene expression was determined by gene-expression arrays (Affymetrix HGU133 Plus 2.0 GeneChips). These samples were normalized, managed and analyzed by GraphPad Prism 5 Software using Spearman correlation coefficients. Detailed patient characteristics are described in Supplementary Table 2.

### In vivo leukemogenesis assays

C57BL/6J mice, athymic nude mice, and NOD/SCID/γ_c_^null^ immunodeficient NOG mice (4–6 weeks old, male) were purchased from Harlan Laboratories (Madison, WI) and Charles River (Beijing, China). All animal experiments were approved by the Institutional Animal Care and Use Committees of University of Minnesota and the Chinese PLA General Hospital, and were performed in accordance with the U.S. National Institutes of Health (NIH) Guide for Care and Use of Laboratory Animals. NOD/SCID/γ_c_^null^ immunodeficient NOG mice were maintained under specific pathogen-free conditions at the animal facility of Chinese PLA General Hospital.

For the xenograft mouse model (solid tumor-like), SKNO-1 PGK cells were transfected with EZH1 shRNA, and about 3 × 10^6^ transfected cells were injected subcutaneously into the bilateral flanks of each mouse (*n* = 3 mice/group). Tumor diameters were measured with digital caliper; and tumor volume was calculated using the formula: tumor volume (mm^3^) = (length × width × height × π/6). Mice were sacrificed at day 56 after the leukemia cell inoculation and the tumors were harvested. The samples were immediately fixed in 10% neutral-buffered formalin and processed for H&E and IHC staining.

For the leukemic xenograft mouse model, Kasumi-1 and SKNO-1 PGK cells were infected with EZH1 shRNA or scrambled retrovirus. The NOD/SCID/γ_c_^null^ immunodeficient NOG mice were irradiated at 2.5 Gy. After confirming changes in EZH1 expression, about 1–3 × 10^6^ infected cells were injected into the irradiated mice through the tail vein (*n* = 5 mice/group) 24 h after irradiation. Mononuclear cells collected from bone marrow, blood, and spleen of euthanatized mice were stained with mouse anti-human CD45, CD19, and CD33 monoclonal antibodies, as well as rat anti-mouse CD45 (all from BD Pharmingen). The presence of a CD45^+^CD33^+^CD19^−^ population was considered as leukemia engraftment. The number of human leukemic cells was calculated by the equation: total cell number × % of human CD45^+^CD33^+^CD19^−^ cells. The samples were immediately fixed in 10% neutral-buffered formalin and processed for H&E and IHC staining.

For mouse models bearing leukemic disease, a total of 1 × 10^7^ C1498 cells, a murine myelogenous leukemia cell line, were mixed with 15 μg of AML1-ETO and/or EZH1 expression plasmids and electroporated in a 0.4 cm cuvette (Bio-Rad Laboratories, Inc., Hercules, CA) using a Bio-Rad Gene Pulser II™ set to 250 V, 950 µF. The total amount of the transfected DNA was kept constant by adding a corresponding empty vector. After 48 h, about 0.1 × 10^6^ of viable cells were injected into the tail vein of each C57BL/6J mouse. Mice were sacrificed on day 21 after injection. Cytospin preparations of bone marrow cells were processed for Giemsa staining. The lungs, spleens, and livers were harvested and immediately fixed in 10% neutral-buffered formalin and stained with H&E.

For leukemogenic mouse model, bone marrow cells were isolated from the donor C57BL/6J mice (6–8 weeks old, male) pretreated with 5-fluorouracil (250 mg kg^−1^). The retroviral plasmid vectors pMSCV-AML1-ETO (or AML1-ETOK43R, or FLT3-ITD), pVSV, and pGAG-POL were cotransfected into the 293 EBNA packaging cell line using Lipofectamine reagents. To establish BM cells expressing AML1-ETO plus FLT3-ITD or AML1-ETOK43R plus FLT3-ITD, BM cells were infected with AML1-ETO plus FLT3-ITD or AML1-ETOK43R plus FLT3-ITD viruses for 2 days in transplant medium. Then about 5 × 10^5^ transduced and nontransduced BM cells (if less than 5 × 10^5^ transduced cells per recipient were available) were injected through the tail vein into the lethally-irradiated recipient C57BL/6J mice (8–10 weeks old; male; 2 × 4.5 Gy, 3 to 4 h between each dose). Recipient mice were monitored weekly for signs of leukemia beginning on day 7 after transplantation. Peripheral blood or bone marrow was prepared and stained with phycoerythrin-conjugated or allophycocyanin-conjugated mouse antibodies (BD Pharmingen) for flow cytometry using established methods^3,39^. Supplementary Fig. 1k is for FACS sequential gating strategy.

### AML patient samples

This study was approved by the Institutional Review Board of the Chinese PLA General Hospital and conducted in accordance with the Declaration of Helsinki. The diagnoses of acute myeloid leukemia (AML) and chronic myeloid leukemia (CML) were made according to the criteria of World Health Organization. Mononuclear cells from BM samples of 122 newly diagnosed, untreated AML patients, and 4 healthy donors were prepared by Ficoll-Hypaque (Sigma-Aldrich, St Louis, MO) gradient centrifugation. AML patient cells were frozen in 10% DMSO plus 90% FBS and cultured in RPMI1640 with 20% FBS. Detection of t(8;21) was routinely performed by standard cytogenetic techniques and/or by FISH using a commercially available AML1-ETO probe (Vysis Inc.). Molecular genetic analysis for AML1-ETO transcripts was conducted using RT-PCR. All patients signed an informed consent document approved by the Institutional Review Board before entering any study. Patient characteristics are summarized in Supplementary Table 1.

### Statistical analysis

SPSS 15.0 software was used to process the data. The Wilcoxon signed-rank test was performed to determine the difference in the expression of AE and EZH1 among the clinical samples. The sample sizes for each study were chosen to be sufficient to allow statistical analysis of the outcomes of the experimental versus control of the studies based on literature documentation of similar well-characterized experiments. In vitro experiments, such as qPCR, cell proliferation assays, Western blotting, etc. were routinely repeated three times unless indicated otherwise in Fig legends or main text. The statistical analysis was performed using the Student’s *t* test. All analyses were performed using the GraphPad Prism 5 Software. *P* *<* 0.05 was considered statistically significant. All *P* values were two-tailed. No samples or animals were excluded from the analysis. All criteria were pre-established. No randomization was used in our studies. No blinding for all experiments. Variations were compatible between groups.

To compare clinical outcomes of patients with different EZH1 expression levels, the cohort was stratified using the quartile grouping method. All AML1-ETO patients were grouped into quartiles according to EZH1 expression levels (Q1-Q4, each quartile containing 25% of the patients) and divided into high EZH1 (Q4; *n* = 16) and low EZH1 (Q1-Q3; *n* = 46) based on the trend observed in clinical outcome after performing a Cox Regression analysis for EFS with EZH1 quartile grouping as the independent variable. Survival curves were generated using the Kaplan–Meier method and the log-rank test was used to compare survival between groups. Clinical features across groups were compared using the 2-sided Fisher exact test for categorical data, and the nonparametric Mann–Whitney *U* test for continuous variables. The Student’s *t* test was performed to compare gene overexpression-induced or knockdown-induced changes against respective controls. A *P* value of less than 0.05 was chosen as the threshold for statistical significance. A full data will be made available to public access.

### Reporting summary

Further information on research design is available in the Nature Research Reporting Summary linked to this article.

## Supplementary information

Supplementary Information

Description of Additional Supplementary Files

Supplementary Data 1

Supplementary Data 2

Supplementary Data 3

Supplementary Data 4

Reporting Summary

Source Data

## Data Availability

All the RNA-Seq data used in this paper are deposited in the Bioproject under the BioProject ID 397611, and the ChIP-Seq data in the Bioproject under the BioProject ID PRJNA298516 and 397609. The proteomics data is uploaded to iProx database with identifier IPX0001773001 (http://www.iprox.org/index). All other relevant data supporting the key findings of this study are available within the article and its Supplementary Information files or from the corresponding authors upon reasonable request.The source data underlying Figs. 1a–d, g–h, l, n, 2a–d, 3a–e, g, 4a–g, i, 5c–I, 6a-b, f, 6h and Supplementary Figs. 1e, g, h–j, 2a–c, 3a, b, d–e, 4a–k, m–o, 5b–l, 6a and c are provided as a Source Data file. A reporting summary for this Article is available as a Supplementary Information file.
