# Supplementary material for: Protein lysine 43 methylation by EZH1 promotes AML1-ETO transcriptional repression in leukemia
Source: Nat Commun. 2019 Nov 7;10:5051. doi: 10.1038/s41467-019-12960-6 (PMC6838331; doi:10.1038/s41467-019-12960-6)
Supplement: Supplementary file 1 — Supplementary Information [file 41467_2019_12960_MOESM1_ESM.pdf]

# Protein Lysine 43 Methylation by EZH1 Promotes AML1-ETO Transcriptional Repression in Leukemia

**Dou *et al***

**Supplementary Figures and Figure Legends**

**Supplementary Tables**

**Supplementary Methods and Parameters for Proteomic Analysis**

## Supplementary Figures and Figure Legends

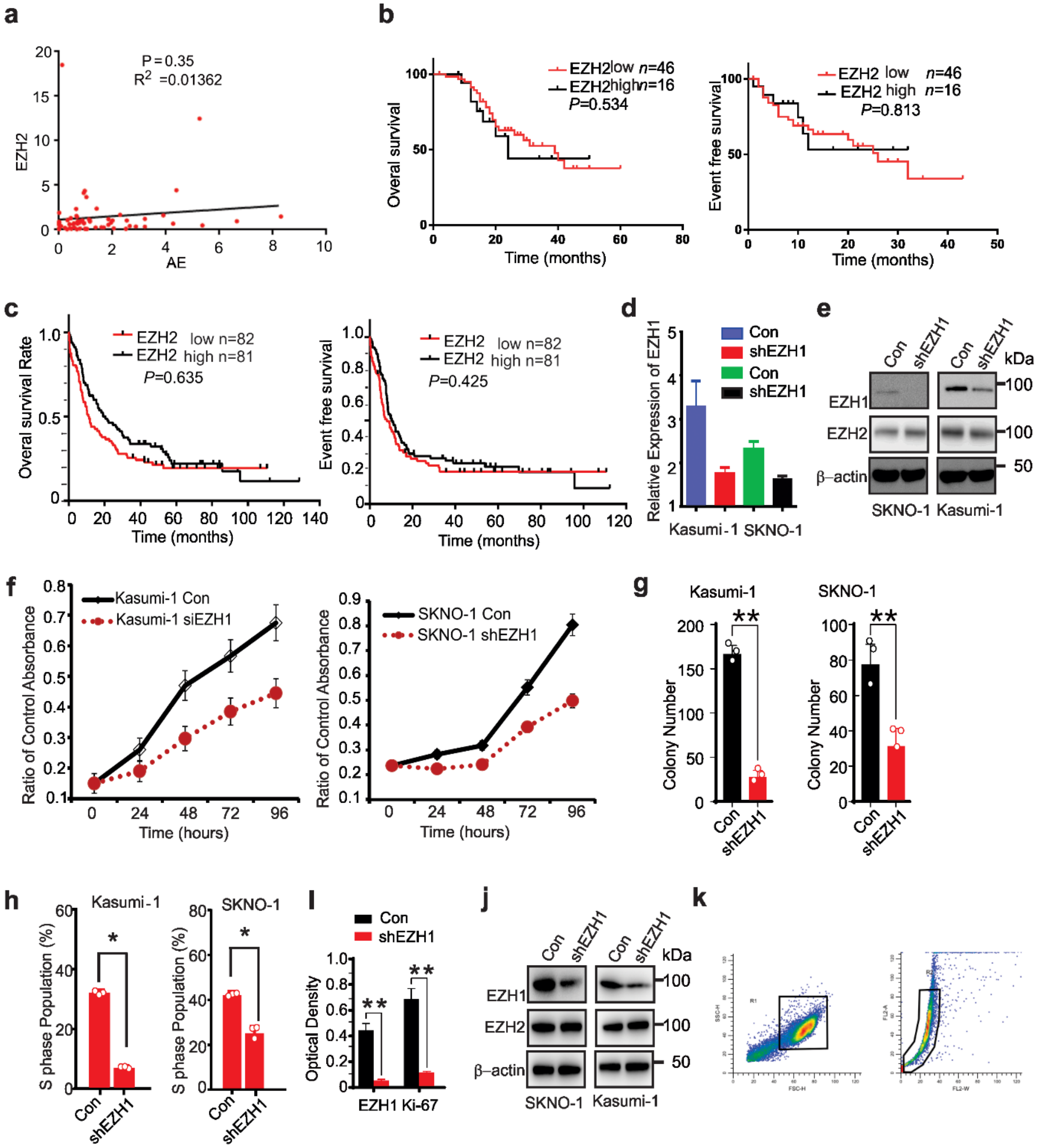


**Supplementary Figure 1. The role for EZH1/2 in AML1-ETO-positive leukemia cells.** (a) Correlation analysis between AML1-ETO and EZH2 mRNA levels in AML1-ETO-positive leukemia patients (n = 62). R: Pearson correlation coefficients; $R^2$: indicates "the goodness of

fit". Statistical significance was calculated by Pearson correlation coefficients. (**b**) The association of EZH2 expression in AML1-ETO-positive patients (n = 62; from Fig. 1**c**) with patient overall survival (OS) and event free (EFS) survival was analyzed using the Kaplan–Meier estimate. (**c**) The association of EZH2 expression with patient OS and EFS survival in primary samples of 347 patients with *de novo* AML (GSE6891) was analyzed using the Kaplan–Meier estimate (n = 347). (**d**) qPCR and analysis for mRNA levels of EZH1 in Kasumi-1 and SKNO-1 cells transfected with vehicle (Con) or EZH1 shRNA vectors (n = 3). (**e**) Western blotting to detect protein levels of EZH1 and EZH2 in Kasumi-1 and SKNO-1 cells transfected with scrambled (Con) or EZH1 shRNA (shEZH1) vectors. (**f**) Growth curves using CCK-8 assays for Kasumi-1 and SKNO-1 cells transfected with scrambled or EZH1 shRNA vectors. Data are expressed as mean values ± S.D. from 3 independent experiments. (**g**) Colony-forming analysis for Kasumi-1 (left) and SKNO-1 (right) cells transfected with scrambled (con) or EZH1 shRNA (shEZH1) vectors (n = 3). (**h**) Cell cycle analysis for Kasumi-1 and SKNO-1 cells transfected with scrambled (Con) or EZH1 shRNA (shEZH1) vectors (n = 3). Data are expressed as mean values ± S.E.M. of duplicate samples from three independent experiments; (**i**) Histogram showing quantification of the immunohistochemical signal from Figure 1**i** (n = 3). (**j**) Western blotting in Kasumi-1 and SKNO-1 cells infected with scrambled (Con) or EZH1 shRNA (shEZH1) viruses for 24 hours and selected by 2 µg/ml puromycin for additional 48 hours. (**k**) Gating strategy to determine the cell cycle from **h**. Data are expressed as mean values ±S.D; $^{**}P < 0.001$, $^{*}P < 0.05$; Figure **g**, **h**, **I**, one-way ANOVA; Figure **b**, **c**, log-rank test; Source data are included in the Source Data file.

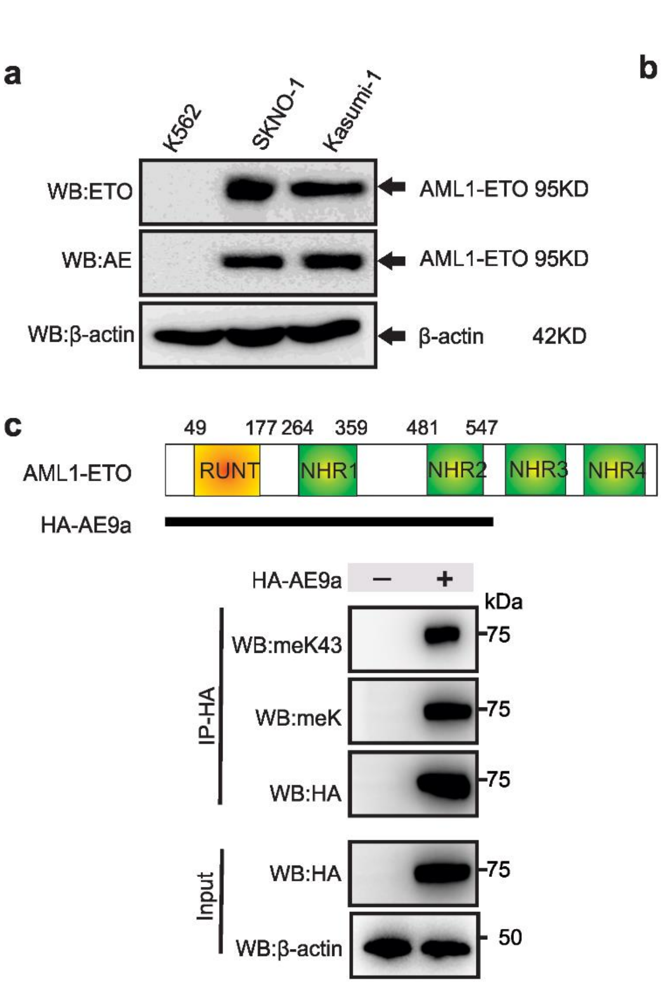


**Supplementary Figure 2. Lysine residues of the AML1-ETO protein are methylated.** (**a**) K562, SKNO-1 and Kasumi-1 total cell lysates were subjected to Western blotting. AE, AML1-ETO. (**b**) HEK293 cells were transfected for 48 hours with empty or HA-AE-W expression vectors, and the anti-HA pull-down proteins were subjected to Western blotting. (**c**) Upper: diagram of the AML1-ETO9a (AE9a) construct; lower: HEK293 cells were transfected with HA-AE9a expression or control vector for 48 hours. The anti-HA pull-down and total cell lysates were subjected to Western blotting.

Note: IP, Immunoprecipitation; WB, Western blotting; AE, AML1-ETO; meK, commercial lysine methylation antibody; meK43, customized methylated AEK43 antibody; The “Input” of

each panel is the immunoblot analysis for whole cell extracts to show the target protein levels. The data are representative of 3 independent experiments. Source data are included in the Source Data file.

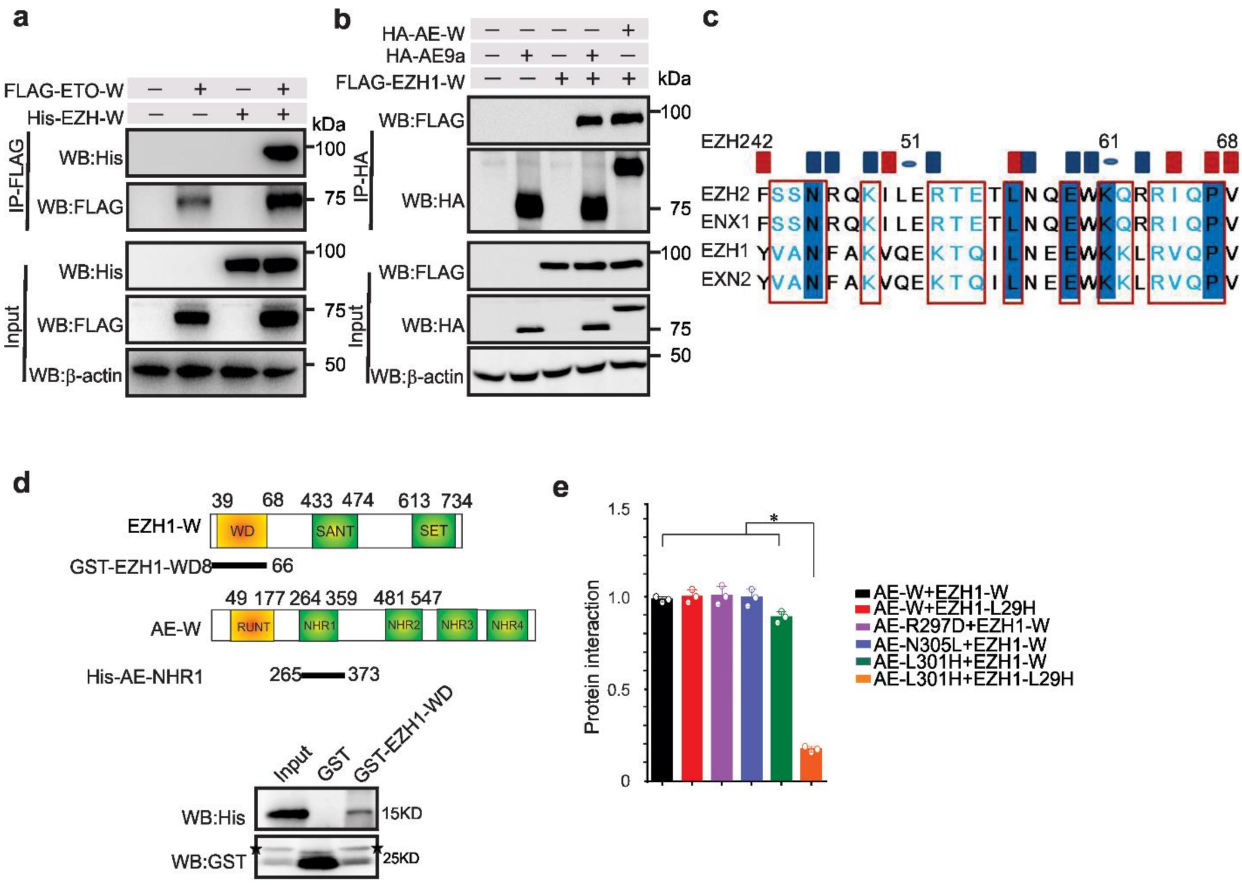


**Supplementary Figure 3. AML1-ETO and EZH1 have physical interaction.** (**a**) The anti-FLAG immunoprecipitates (IP) and whole cell lysates (Input) from HEK293 cells expressing FLAG-ETO-W, His-EZH1-W or both were subjected to Western blotting. (**b**) Western blotting for anti-HA immunoprecipitates and total cell lysates from HEK293 cells expressing HA-AE-W or HA-AE9a alone or plus FLAG-EZH1-W. (**c**) Sequence alignment of the WD domain of EZH2 with those of EZH1, ENX1 and ENX2. Residues involved in intermolecular hydrogen bonds/salt bonds and van der Waals contacts are indicated by red and blue squares on top. (**d**) Upper: Schematic representation of GST and GST-EZH1-WD and/or His-AE-NHR1; Lower: *Escherichia coli BL21*-expressing GST or GST-EZH1-WD plus His-AE-NHR1 were purified. Equal amounts of purified GST-EZH1-WD proteins were incubated with the *in vitro* His-AE-

NHR1 protein, and then the GST pulled-down complexes were subjected to Western blotting using anti-His or anti-GST. (**e**) Quantification of protein interaction. HEK293 cells were transfected for 48 hours with HA-AE-W (wild type) and FLAG-EZH1-W (wild type) or the indicated mutant constructs (n = 3). The anti-FLAG immunoprecipitates were subjected to Western blotting. Data are expressed as mean values ±S.D; **P* < 0.05; Figure **e**, one-way ANOVA.

Note: IP, Immunoprecipitation; WB, Western blotting; AE, AML1-ETO. The "Input" of each figure is the immunoblot analysis for whole cell extracts to show the target protein levels. The data are representative of 3 independent experiments. Source data are included in the Source Data file.

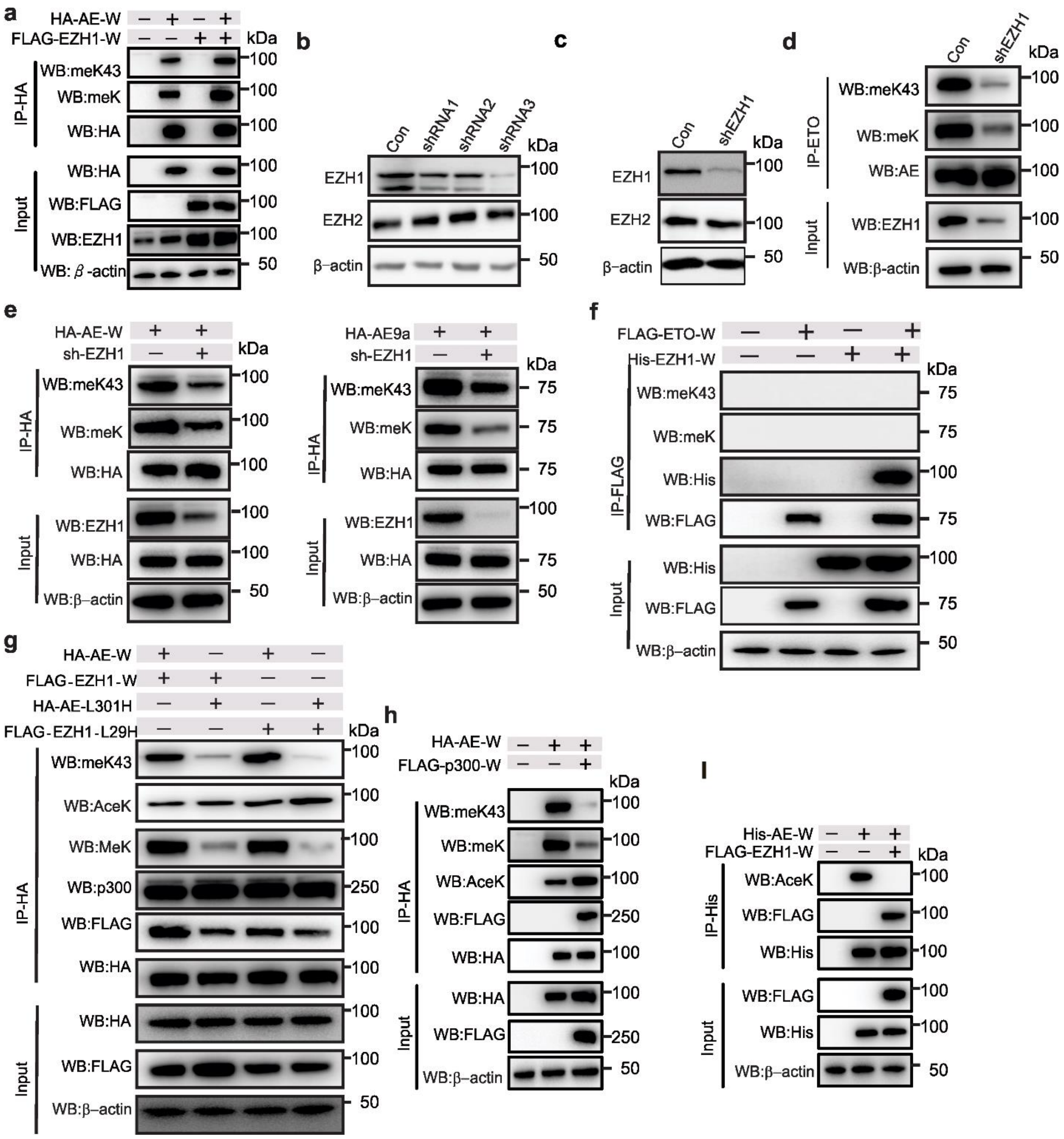
a
HA-AE-W
FLAG-EZH1-W
kDa
IP-HA
WB:meK43
WB:meK
WB:HA
Input
WB:HA
WB:FLAG
WB:EZH1
WB: β-actin
100
50
b
Con
shRNA1
shRNA2
shRNA3
kDa
EZH1
EZH2
β–actin
c
Con
shEZH1
EZH1
EZH2
β–actin
d
Con
shEZH1
IP-ETO
WB:meK43
WB:meK
WB:AE
Input
WB:EZH1
WB:β-actin
e
HA-AE-W
sh-EZH1
HA-AE9a
WB:EZH1
WB:β–actin
75
f
FLAG-ETO-W
His-EZH1-W
IP-FLAG
WB:His
WB:FLAG
g
FLAG-EZH1-W
HA-AE-L301H
FLAG-EZH1-L29H
WB:AceK
WB:MeK
WB:p300
250
h
FLAG-p300-W
i
His-AE-W
IP-His

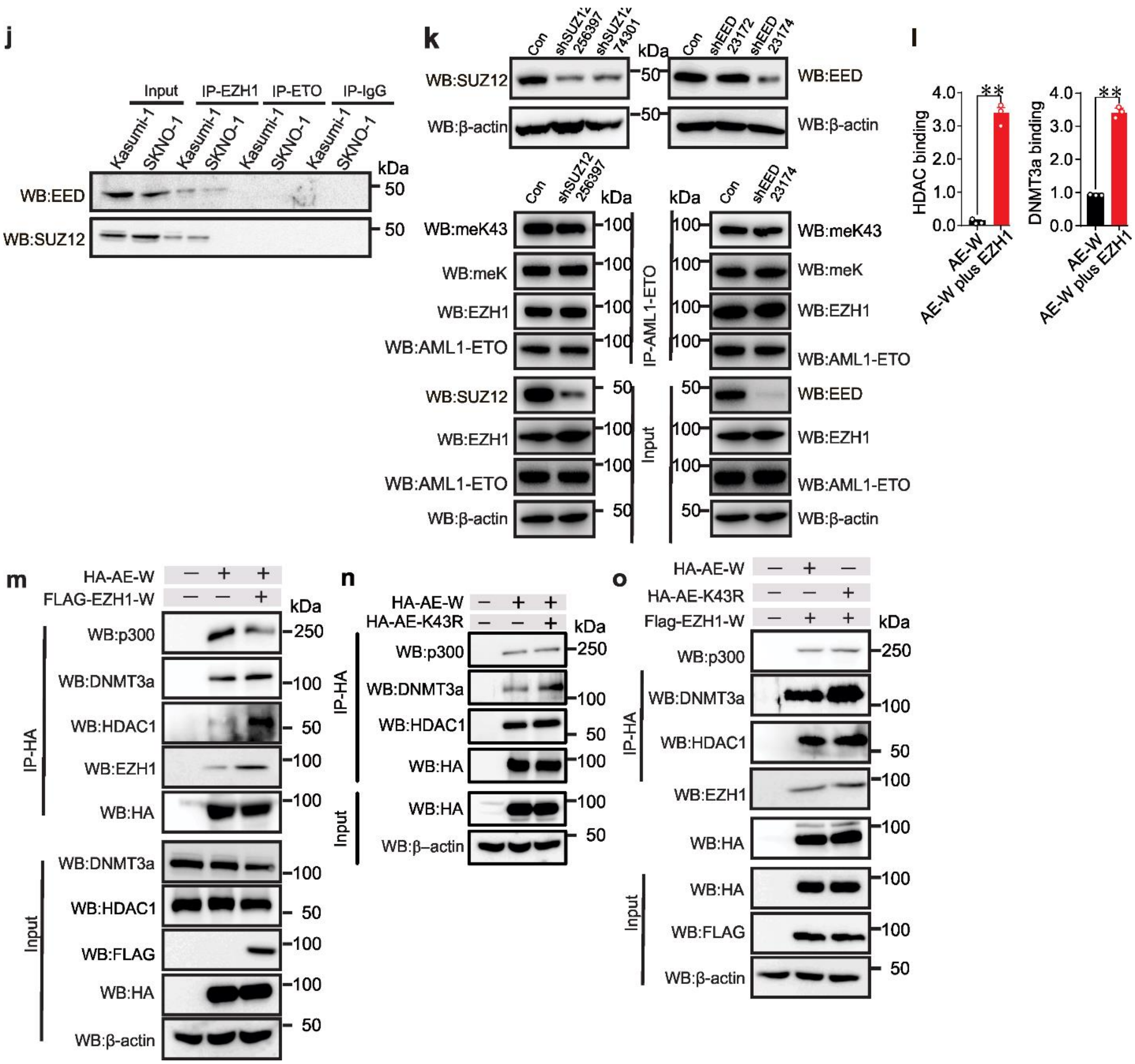


**Supplementary Figure 4. EZH1 methylates AML1-ETO at Lys43.** (**a**) HEK293 cells were transfected with HA-AE-W alone or HA-AE plus FLAG-EZH1-W. The anti-HA immunoprecipitates were subjected to Western blotting. (**b**) Characterization of EZH1 shRNA knockdown efficacy. (**c**) The knockdown of EZH1 was verified by Western blotting. EZH2 was not affected. (**d**) SKNO-1 cells were transfected with EZH1 or scrambled shRNA vectors. Endogenous AML1-ETO was immunoprecipitated by anti-ETO followed by probing with anti-

meK and anti-meK43. (**e**) HEK293 cells were transfected with HA-AE-W (left), HA-AE9a (right) alone or plus EZH1 shRNA3. The anti-HA immunoprecipitates were subjected to Western blotting. (**f**) HEK293 cells were transfected with either FLAG-ETO-W alone or plus His-EZH1-W. ETO proteins immunoprecipitated by anti-FLAG were subjected to Western blotting. (**g**) HEK293 cells were transfected with HA-AE-W and FLAG-EZH1-W or the indicated mutant constructs. The anti-HA immunoprecipitates were subjected to Western blotting. (**h**) HEK293 cells were transfected with HA-AE-W alone or HA-AE-W plus FLAG-p300 for 48 hours and AML1-ETO pull-down by anti-HA was subjected to Western blotting. (**i**) HEK293 cells were transfected with His-AE-W alone or His-AE-W plus FLAG-EZH1-W, AML1-ETO pull-down by anti-His was subjected to Western blotting (n = 3). (**j**) Anti-ETO or anti-EZH1 immunoprecipitates from Kasumi-1 and SKNO-1 cells were subjected to Western blotting. (**k**) Upper, Kasumi-1 cells were transfected with different EED or SUZ12 shRNAs or scrambled vectors and the knockdown efficiency was verified by Western blotting. The efficient shRNAs, shSUZ256397 for SUZ12 and shEED23174 for EED were selected for further investigations. Lower, the AML1-ETO protein was pulled down by an AML1-ETO specific antibody and subjected to Western blotting in Kasumi-1 cells with EED or SUZ12 knockdown. (**l**) Graph illustrating the quantification of DNMT3a and HDAC1 binding to AML1-ETO after the EZH1 introduction shown in Supplementary Figure 4**m**. (**m**) Western blotting for anti-HA immunoprecipitates from HEK293 cells transfected with HA-AE-W alone, or HA-AE-W plus FLAG-EZH1-W. (**n**) Western blotting for anti-HA immunoprecipitates from HEK293 cells expressing either HA-AE-W or HA-AEK43R. (**o**) HEK293 cells were transfected for 48 hours with indicated constructs. Anti-HA immunoprecipitates were subjected to Western blotting.

Note: IP, Immunoprecipitation; WB, Western blotting; AE, AML1-ETO. W, wild type; meK, commercial lysine methylation antibody; meK43, customized methylated AEK43 antibody. The "Input" for each figure is the immunoblot analysis of whole cell extracts to demonstrate the target protein levels. Data are expressed as mean values ±S.D; $^{**}P < 0.001$; Figure **i**, one-way ANOVA. The data are representative of 3 independent experiments. Source data are included in the Source Data file.

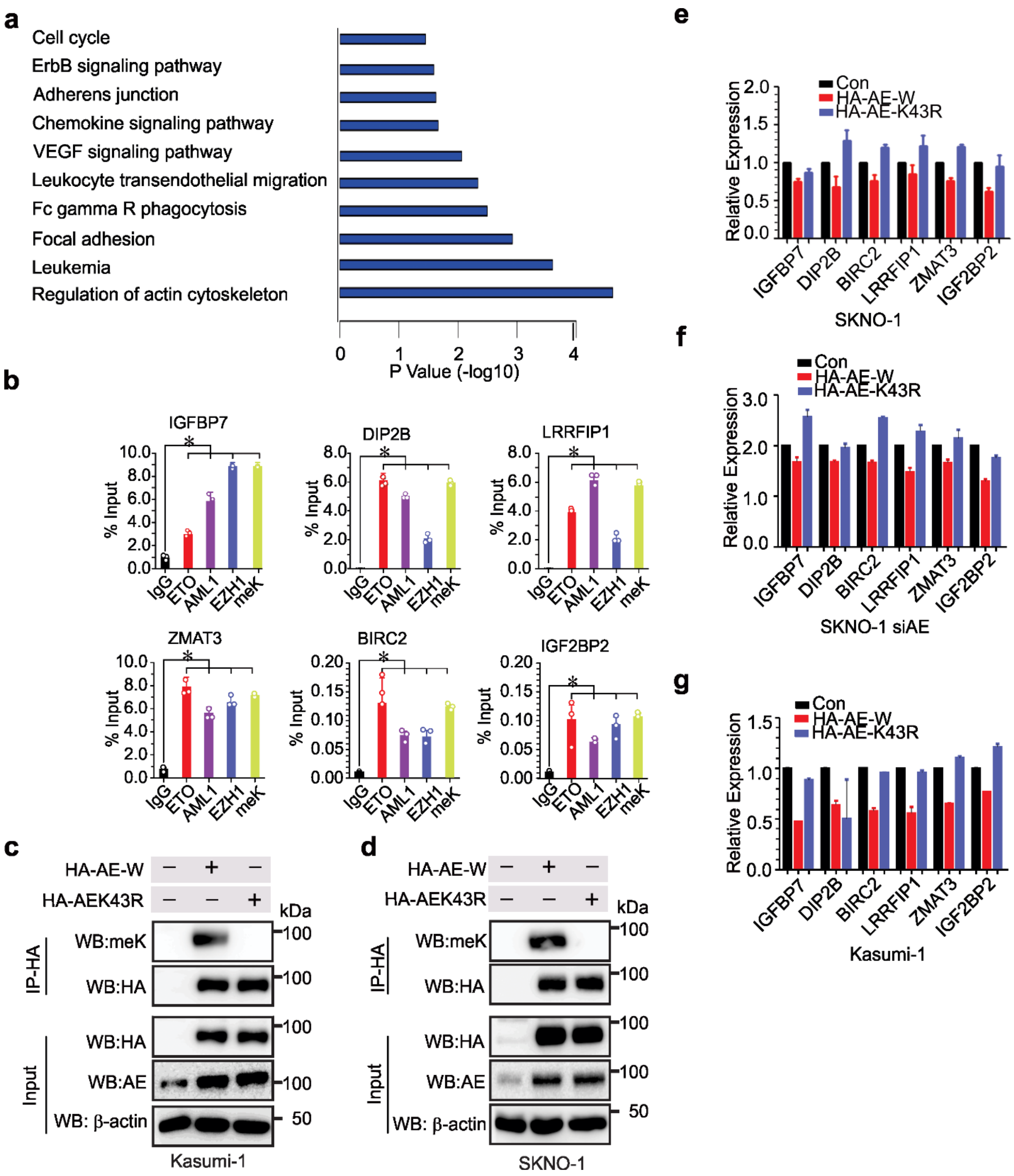
a
Cell cycle
ErbB signaling pathway
Adherens junction
Chemokine signaling pathway
VEGF signaling pathway
Leukocyte transendothelial migration
Fc gamma R phagocytosis
Focal adhesion
Leukemia
Regulation of actin cytoskeleton
0 1 2 3 4
P Value (-log10)
b
IGFBP7
DIP2B
LRRFIP1
ZMAT3
BIRC2
IGF2BP2
% Input
IgG ETO AML1 EZH1 meK
c
HA-AE-W
HA-AEK43R
kDa
IP-HA
WB:meK
WB:HA
Input
WB:HA
WB:AE
WB: β-actin
100
50
Kasumi-1
d
HA-AE-W
HA-AEK43R
kDa
IP-HA
WB:meK
WB:HA
Input
WB:HA
WB:AE
WB: β-actin
100
50
SKNO-1
e
Con
HA-AE-W
HA-AE-K43R
Relative Expression
IGFBP7 DIP2B BIRC2 LRRFIP1 ZMAT3 IGF2BP2
SKNO-1
f
Con
HA-AE-W
HA-AE-K43R
Relative Expression
IGFBP7 DIP2B BIRC2 LRRFIP1 ZMAT3 IGF2BP2
SKNO-1 siAE
g
Con
HA-AE-W
HA-AE-K43R
Relative Expression
IGFBP7 DIP2B BIRC2 LRRFIP1 ZMAT3 IGF2BP2
Kasumi-1

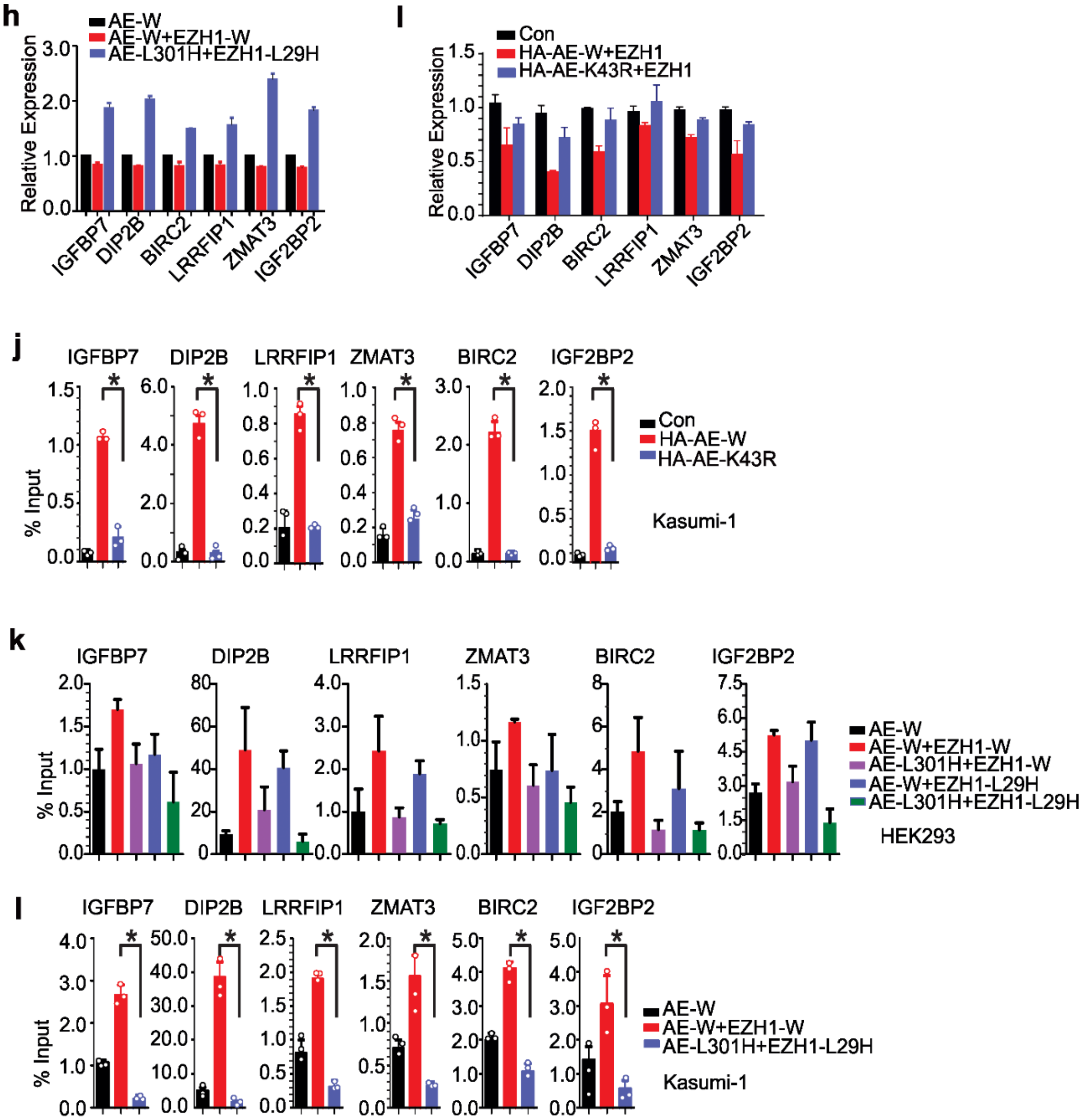


**Supplementary Figure 5. Identification of target genes for both AML1-ETO and EZH1.** (**a**) Enrichment scores for Gene Ontology (GO) categories in a set of AML1-ETO and EZH1 ChIP-Seq common target genes. The –log (*P* value) axis indicates the statistical significance of the functions to the dataset. (**b**) ChIP-qPCR showing the co-occupancy of AML1-ETO, EZH1 and meK (commercial lysine methylation antibody) in AML1-ETO target promoters in Kasumi-1

cells determined by using anti-AML1, anti-ETO, anti-EZH1 or anti-meK (commercial lysine methylation antibody) (n = 3). (**c**, **d**) Western blotting for anti-HA immunoprecipitates from Kasumi-1 (**c**) and SKNO-1 (**d**) cells expressing HA-AE-W (lane 2) or HA-AEK43R (lane 3). meK, commercial lysine methylation antibody. (**e**, **f**, **g**) qPCR for changes in AML1-ETO and EZH1 target gene expression in SKNO-1 (**e**), SKNO-1 siAE (**f**) and Kasumi-1 (**g**) cells transfected with AE-W or AEK43R (n = 3). (**h**, **i**) qPCR for changes in AML1-ETO and EZH1 target gene expression in HEK293 cells transfected with AE-W alone, AE-W plus EZH1-W or AEL301H plus EZH1L29H (n = 3). (**j**, **k**) ChIP-qPCR assessing the enrichment of AE in the target promoters in Kasumi-1 cells (**j**) transfected for 48 hours with HA-AE-W or HA-AEK43R, and in HEK293 cells (**k**) transfected for 48 hours with HA-AE-W and FLAG-EZH1-W or indicated mutant constructs (n = 3). (**l**) ChIP-qPCR assessing the enrichment of AE and its mutants on the target promoters in Kasumi-1 cells transfected for 48 hours with HA-AE-W and FLAG-EZH1-W or indicated mutant constructs (n = 3).

Note: AE, AML1-ETO; W, wild type. The data are representative of 3 independent experiments and data are expressed as mean values ± S.D; *$P < 0.05$; Figure **b**, **j**, **i**, one-way ANOVA. Source data are included in the Source Data file.

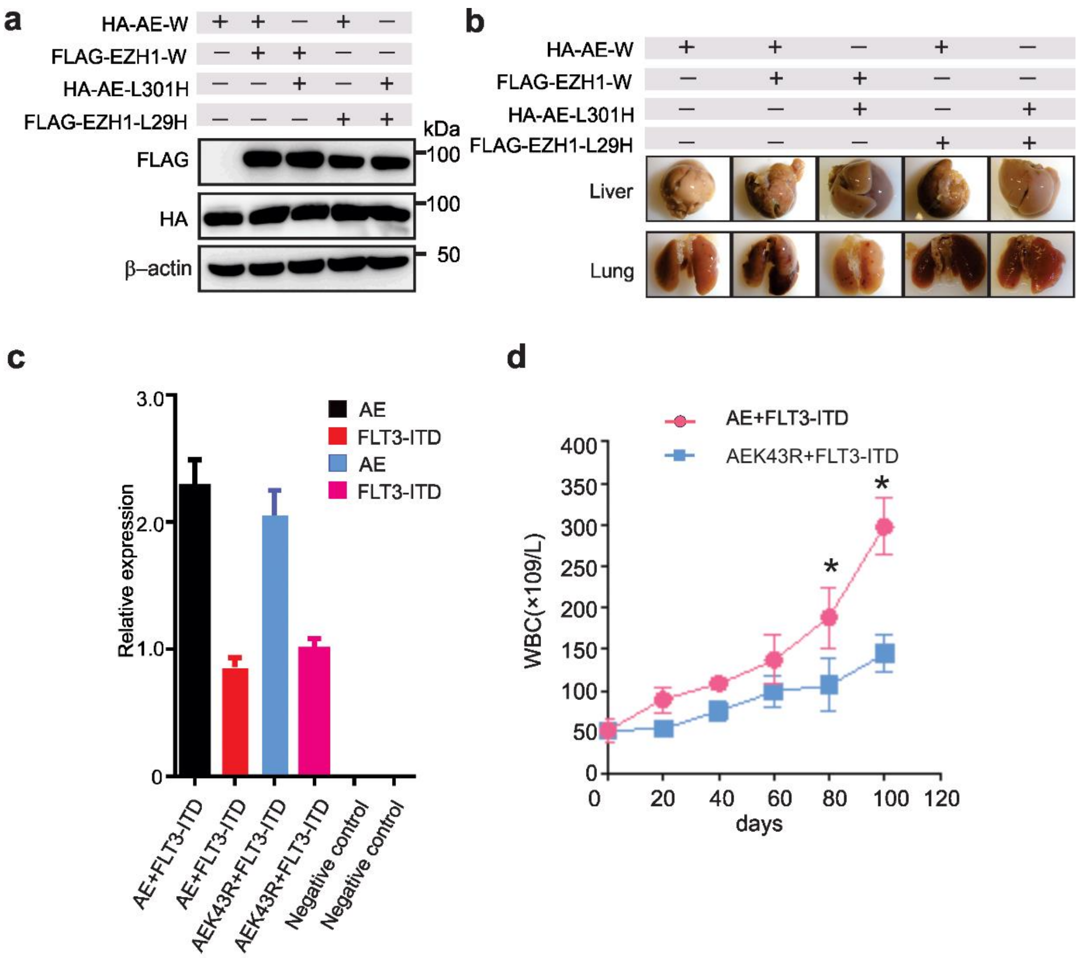


**Supplementary Figure 6. EZH1-mediated Lys43 methylation is essential for leukemic cell expansion.** (**a**) C1498 cells were transfected with HA-AE-W (wild type) and FLAG-EZH1-W (wild type) or indicated mutant constructs, and the whole cell lysates were subjected to Western blotting. (**b**) Representative external views of the livers and lungs isolated from C57BL/6J mice (n = 3 mice/group) injected with C1498 cells transfected with HA-AE-W (wild type) and FLAG-EZH1-W (wild type) or the indicated mutant constructs. (**c**) Bone marrow cells were transduced with AML1-ETO plus FLT3-ITD or AML1-ETOK43R plus FLT3-ITD viruses, and the expression of indicated gene was analyzed by qPCR (n = 3). (**d**) White blood cell (WBC)

analysis of peripheral blood from engrafted mice with AML1-ETO plus FLT3-ITD or AML1-ETOK43R plus FLT3-ITD (n = 5).

Note, Figure **a**, **c**, data are representative of 3 independent experiments. Data are expressed as mean values ±S.D; *$P < 0.05$; Figure **c**, **d**, one-way ANOVA. Source data are included in the Source Data file.

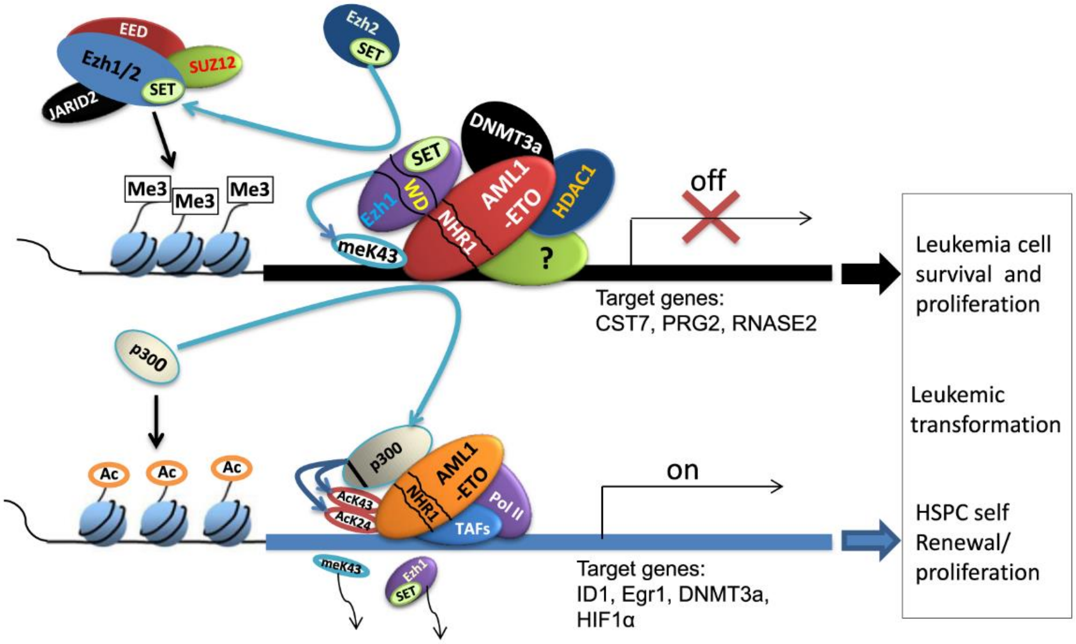


**Supplementary Figure 7. Schematic model illustrating the role of EZH1 in facilitating AML1-ETO-induced transcriptional activities and leukemogenicity.** Top view illustrates that EZH1 directly interacts with AML1-ETO at the target gene loci. The recruitment of EZH1 is required for AML1-ETO Lys43 methylation, transcriptional repression and leukemia-promoting property. The introduction of EZH2 dissociates EZH1 from AML1-ETO to shape the PRC2 complex for controlling H3K27Me3. The lower view shows that p300 recruitment disrupts the EZH1 interaction with and Lys43 methylation of AML1-ETO, but mediates lysine acetylation of Lys43 and Lys24 resulting in the activation of additional target genes followed by HSPC self-renewal/proliferation or leukemic transformation. HSPC, hematopoietic stem/progenitor cell.

## Supplementary Tables

### Supplementary Table 1: Clinical characteristics of AML patients

| Characteristic | AE-positive Patients | AE-negative Patients |
|---|---|---|
| N | 62 | 60 |
| Age | 38 (17-62) | 29 (11-64) |
| Sex, N | | |
| Male | 23 | 26 |
| Female | 39 | 34 |
| WBC ($\times 10^9$/L) [a] | 26.1 (2.2-66.0) | 13.9 (1.9-61.8) |
| BM blasts (%) [a] | 64.9 (10.0-97.0) | 29.3 (42.0-87.0) |
| C-KIT mutation status b, N (%) | 11 (17.7%) | |
| HSCT, N (%) | | |
| Allo-HSCT | 10 (16.1%) | 8 (13.3%) |
| Auto-HSCT | 7 (11.3%) | 2 (3%) |
| CR [b], no. (%) | | |
| 1 course | 47 (75.8) | 40 (67.7%) |
| ≥ 2 courses | 15 (24.2) | 20 (33.3%) |

**Supplementary Table 2: Clinical characteristics of AML patients of GEO database GSE6891**

| Variable | Patients (N) | Median age, y (range) | Sex (male/female) |
|---|---|---|---|
| Total | 347 | 43 (15-61) | 190/157 |
| Cytogenetic status | | | |
| t(8;21) | 35 | 37 (16-54) | 21/14 |
| -5/7(q) | 20 | 45 (15-68) | 12/8 |
| -9q | 8 | 51 (34-63) | 3/5 |
| 11q23 | 8 | 45 (30-66) | 4/4 |
| Complex | 8 | 44 (18-59) | 4/4 |
| inv(16) | 26 | 38 (16-74) | 14/12 |
| NN | 157 | 48 (18-77) | 73/84 |
| t(15;17) | 7 | 37 (22-54) | 4/3 |
| t(9;22) | 2 | 31 (30-32) | 1/1 |
| trisomy 8 | 14 | 50 (20-71) | 9/5 |
| Others | 62 | 49 (17-74) | 31/31 |

**Supplementary Table 3: Univariate and multivariate analyses for OS and EFS in 122 AML patients (Refer Fig. 1c)***

| | Univariate | | Multivariate | |
|---|---|---|---|---|
| | OS | EFS | OS | EFS |
| NR post-first induction therapy | 4.723 (2.134-9.930) | 3.966 (1.787-8.803) | — | — |
| NR post-second induction therapy | 10.378 (5.129-23.026) | 13.722 (6.173-23.557) | — | — |
| No Extramedullary infiltration | 0.398 (0.172-0.832) | 0.298 (0.167-0.712) | 0.078 (0.023-0.398) | 0.178 (0.047-0.398) |
| Age (≥60 cf. <60 y) | 3.177 (1.085-9.137) | 3.912 (1.446-10.986) | — | 5.252 (2.421-28.996) |
| ECOG score ≥3 | 3.953 (1.081-15.955) | | 15.51 (2.598-83.57 | — |
| EZH1 high expression | 2.711 (1.253-6.823) | 3.012 (1.113-7.554) | — | 3.599 (1.884-7.936) |
| C-KIT mutation | 2.277 (1.053-9.335) | 2.989 (1.956-10.223) | — | 3.133 (1.077-8.996) |

** Reported as HR, 95% CI*

*Abbreviations: WBC, white blood cell; BM, bone marrow; HSCT, hematopoietic stem cell transplantation; CR, complete remission; NR, Non response*

[a]Indicates data expressed as median (range). [b]Information is not available in some cases.

**Supplementary Table 4: Primer sequences used in the experiments**

| Name (Genebank Accession No.) | | Primer Sequence (5' to 3') |
|---|---|---|
| AE domain truncated (NM_001754) Fused to pcDNA4.0 | Forward | GGGGTACCGCCACCATGCGTATCCCCGTAGATGC |
| | Reverse1 | CCGCTCGAGGCGAGGGGTTGTCTCTATGGT |
| | Reverse2 | CCGCTCGAGACCACAGGCTGGGGGCAGCTG |
| | Reverse3 | CCGCTCGAGTGTGCCATGCAACCCCATAGG |
| | Reverse4 | GTGCTTCTCAGTACGATTGCCGCTCCTCAGCTTGC |
| EZH1domain truncated (NM_001991) Fused to pCMV6-entry | Forward1 | GAGGCGATCGCATGGAAATACCAAATCCCCCTACCT |
| | Forward2 | GAGGCGATCGCATGCAGTCAATGAAGCCTGTGAG |
| | Reverse1 | CGACGCGTAAGGACGTCGGTCTCCCTCTC |
| | Reverse2 | CGACGCGTCTTAAGTCCACGCTGGATGCT |
| | Reverse3 | CGACGCGTCACAGGCTCCGAGGGTGCTTCCAC |
| AE K43R mutation (NM_001754) | Forward 1 | CGGCGCTGCCCTGGCCGGCAGGCTGAGGAGCGGCGACCGCAG |
| | Reverse | CTGCGGTCGCCGCTCCTCAGCCTGCCGGCCAGGGCAGCGCCG |
| AE K24R mutation (NM_001754) | Forward 1 | ACCGCGCTGAGCCCAGGCAGGATGAGCGAGGCGTTGCCGC |
| | Reverse | GCGGCAACGCCTCGCTCATCCTGCCTGGGCTCAGCGCGGT |
| AE expression (NM_001754) | Forward | CAAGTCGCCACCTACCACAGA |
| | Reverse | AGCCTAGATTGCGTCTTCACATC |
| EZH1 expression (NM_001991) | Forward1 | TCTGAATACTGTGGTGAG |
| | Reverse1 | TATTGAGGTTGAAGAGGAA |
| | Forward2 | ATACGACTGCTTCCTTCA |
| | Reverse2 | TGGTTCTGGTTCAATCTTG |
| | Forward3 | TCTGAATACTGTGGTGAG |
| | Reverse3 | TATTGAGGTTGAAGAGGAA |
| ABL1 expression (NM_007313) | Forward | CTCCATTATCCAGCCCCAAA |
| | Reverse | TATTGAGGTTGAAGAGGAA |
| AE aa1-aa143 Fused to pGEX-5X-1 | Forward | CGCGGATCCCCATGCGTATCCCCGTAGATGC |
| | Reverse | ATAAGAATGCGGCCGCTTA AGGTGGGGTAGGGTGAGGC |
| EZH1domain truncated (NM_001991) Fused to pGEX-5X-1 | Forward1 | CCGGAATTCATGGAAATACCAAATCC |
| | Reverse1 | ATAAGAATGCGGCCGCTTA AGCATCAGCTTGGCTGTACCTGTAA |
| | Reverse2 | ATAAGAATGCGGCCGCTTAGTGCTTTCTTTTCTTCTTCT |
| | Forward3 | CCGGAATTCGCAGTGCGAGAATGTGAC |
| | Reverse3 | ATAAGAATGCGGCCGCTTA AGCATCAGCTTGGCTGTAC |
| AE NHR1 domain Fused to pET41aHT | Forward | GCGCGCGGATCCGCCTGTGGTGCCAGG |
| | Reverse | GCGCGCCTCGAGTTATGAGGTGGTGCTGGCAT |
| EZH1 WD domain Fused to pET41aHT | Forward | GCGCGCGGATCCACCTCCAAATGTATCACTTAC |
| | Reverse | GCGCGCCTCGAGTTATTGGACACGAAGCTTCTTCCA |
| AE NHR1 domain Fused to pGEX-GST | Forward | GCGCGCGGATCCGCCTGTGGTGCCAGG |
| | Reverse | GCGCGCCTCGAGTTATGAGGTGGTGCTGGCAT |
| EZH1 WD domain Fused to pGEX-GST | Forward | GCGCGCGGATCCACCTCCAAATGTATCACTTAC |
| | Reverse | GCGCGCCTCGAGTTATTGGACACGAAGCTTCTTCCA |

| | | |
|---|---|---|
| IGFBP7 CHIP (NM_001253835) | Forward<br>Reverse | AAACCACAGAGTAATGAG<br>TTAAACTTCTTAACAACCAC |
| IGFBP7 expression (NM_001253835) | Forward<br>Reverse | GAGGCGGAAGGGTAAAGCC<br>GGCACCAGTGACATTCCAGA |
| DIP2B CHIP (NM_ 173602) | Forward<br>Reverse | GTGGTAAAGCCGACAGTCCA<br>CAGTCAGGCTGAAGACGCAT |
| DIP2B expression (NM_173602) | Forward<br>Reverse | GATCCAACAGGTCCAGCCTT<br>TGGCTGAGGTACTTCCACAAT |
| LRRFIP1 CHIP (NM_ 001137550) | Forward<br>Reverse | CAAATCCTGTGCTCACCCCT<br>GACTTTGCGGGGACTGTTTG |
| LRRFIP1 expression (NM_001137550) | Forward<br>Reverse | TGATGAACGGGAATGCTTA<br>TTGCCAATCTTCTGTCTCT |
| ZMAT3 CHIP (NM_022470) | Forward<br>Reverse | AAGTAGTATATTTGTTATCGTTTG<br>AACCTGGACATCTTCAAT |
| ZMAT3 expression (NM_022470) | Forward<br>Reverse | AGGCTCATTATCAGGGTAA<br>GACCACATTGCTCATTCT |
| BIRC2 CHIP (NM_ 001166) | Forward<br>Reverse | AGAATGGTAGACACAGAG<br>GGACTATCCGTATCAACT |
| BIRC2 expression (NM_ 001166) | Forward<br>Reverse | AAGTATATTCCAACAGAAGATG<br>CTACCAGATGACCACAAG |
| IGF2BP2 CHIP (NM_ 001007225) | Forward<br>Reverse | CTCATTTCATGTTGCAGTGTGC<br>ATGACACTCCGGCTAACAGC |
| FLT3 expression (NM_ 004119) | Forward<br>Reverse | GCAATTTAGGTATGAAAGCCAGC<br>CTTTCAGCATTTTGACGGCAACC |
| IGF2BP2 expression (NM_001007225) | Forward<br>Reverse | CTGAGATAGAGATTATGAAGAAG<br>TACAGGCTGGAGAAGTAT |
| HA tagged AML1 expression (NM_001001890.2) | Forward<br>Reverse | 5’-GAATTCGCGATGTACCCATACGATGTTCCAGATTACGCTGC TTCAGACAGC-3’<br>5’-GTGAAGCTTTTCCCTCTTCCACTTCGACCG-3’ |

**Supplementary Table 5: Antibodies used in the experiments**

| Antibody | Application | Company | Catalog No. | Source | Dilution |
|---|---|---|---|---|---|
| DNMT3a | Western blot<br>IHC | Santa Cruz Biotechnology | sc20703 | Rabbit | Western blot:1:500<br>IHC: 1:250 |
| AML1 | Western blot<br>IHC | Cell Signaling Technology | 4334 | Rabbit | Western blot: 1:500<br>IHC: 1:100 |
| EZH1 | Western blot<br>IHC | Thermo Fisher Scientific | PA5-28710 | Rabbit | Western blot: 1:500<br>IHC: 1:100<br>IF 1:300 |
| EZH1 | ChIP<br>CO-IP | Santa Cruz Biotechnology | sc-292275X | Rabbit | CHIP: 5 µg<br>CO-IP: 1:50 |
| Methylated Lysine Antibody | Western blot | Novus Biologicals | NB600-824 | Rabbit | 1:1000 |
| DYKDDDDK Tag Antibody | Western blot<br>CO-IP | Cell Signaling Technology | 2368S | Rabbit | Western blot: 1:500<br>CO-IP: 1:50 |
| β-actin | Western blot | Santa Cruz Biotechnology | sc-1616 | Goat | 1:1000<br>CO-IP: 1:50 |
| ETO | ChIP<br>CO-IP | Santa Cruz Biotechnology | sc-9737 | Goat | CHIP: 5 µg |
| ETO | Western blot | Cell Signaling Technology | 4498s | Rabbit | Western blot: 1:1000 |
| Ezh2 | Western blot<br>CO-IP | Cell Signaling Technology | 5246S | Rabbit | Western blot: 1:500<br>CO-IP: 1:50 |
| HA<br>Tag Antibody | Western blot<br>CO-IP | Cell Signaling Technology | 3724S | Rabbit | Western blot: 1:500<br>CO-IP: 1:50 |
| Histone H3 Antibody (FL-136) | Western blot | Santa Cruz Biotechnology | SC-10809 | Rabbit | Western blot: 1:1000 |
| Tri-methyl-histoneH3(lys27) | Western blot<br>CO-IP | Cell Signaling Technology | 9733S | Rabbit | Western blot: 1:500<br>CO-IP: 1:50 |
| GST (26H1) | Western blot<br>CO-IP | Cell Signaling Technology | 2624 | Mouse | Western blot: 1:500 |
| His-Tag Antibody | Western blot<br>CO-IP | Cell Signaling Technology | 12698 | Rabbit | Western blot: 1:500<br>CO-IP: 1:50 |

| | | | | | |
|---|---|---|---|---|---|
| Di-Methyl-Histone H3 (Lys4) Antibody | Western blot | Cell Signaling Technology | 9726 | Rabbit | Western blot: 1:500 |
| His-Tag | Western blot<br>CO-IP | Cell Signaling Technology | 2366 | Mouse | Western blot: 1:500<br>CO-IP: 1:200 |
| SUZ12 (D39F6) | Western blot<br>CO-IP | Cell Signaling Technology | 3737S | Rabbit | Western blot: 1:500<br>CO-IP: 1:50 |
| EED (H-300) | Western blot<br>CO-IP | Santa Cruz Biotechnology | SC-28701 | Rabbit | Western blot: 1:500<br>CO-IP: 1:50 |
| GST | Western blot | Cell Signaling Technology | 2624 S | Mouse | Western blot: 1:500 |
| His-Tag Antibody | Western blot<br>CO-IP | Cell Signaling Technology | 2366 | Mouse | Western blot: 1:1000<br>CO-IP: 1:200 |
| P300 | Western blot | Santa Cruz Biotechnology | Sc-584 | Rabbit | Western blot:1:500 |
| HDAC1 | Western blot<br>IHC | Abcam | Ab7028 | Rabbit | Western blot:1:500 |
| Acetylated-Lysine Antibody | Western blot | Cell Signaling Technology | 9441 | Rabbit | Western blot: 1:1000 |
| DYKDDDDK Tag Antibody | Western blot | Cell Signaling Technology | 8146 | Mouse | Western blot: 1:500<br>IF 1:1600 |
| Alexa Fluor® 488Anti-Rabbit IgG (H+L) Antibody, | Immunofluorescence | Invitrogen | A11008 | Goat | IF 1:300 |
| Alexa Fluor® 594 nti-Mouse IgG (H+L) | Immunofluorescence | Invitrogen | A21201 | Chicken | IF 1:300 |
| Anti-ETO antibody | Immunofluorescence | Abcam | ab128084 | | IF 1:100 |
| Anti-AML1-ETO antibody | ChIP | GeneTax | GTX60339 | Rabbit | CHIP: 5 µg |
| Anti-p300 antibody | ChIP | Calbiochem | NA-46 | Mouse | CHIP: 5 µg |

**Supplementary Table 6: Plasmids used in the experiments**

| Name of Plasmids | Vectors | Detail Information |
|---|---|---|
| His-AE-W | pcDNA4.0 | AML1-ETO wild type, full length |
| His-AE-ΔNHR1 | pcDNA4.0 | AML1-ETO mutant, without NHR1 domain |
| His-AE-ΔNHR2 | pcDNA4.0 | AML1-ETO mutant, without NHR2 domain |
| His-AE-ΔRUNT | pcDNA4.0 | AML1-ETO mutant, without RUNT domain |
| His-AE-no-NHR | pcDNA4.0 | AML1-ETO mutant, without all NHR domains |
| His-AE-no-NHR2 | pcDNA4.0 | AML1-ETO mutant, with RUNT and NHR1 domains |
| HA-AE-W | pUHD | AML1-ETO wild type, full length, Addgene plasmid #12430 |
| HA-AE-K24R | pUHD | AML1-ETO mutant, Lysine 24 mutated to Arginine |
| HA-AE-K43R | pUHD | AML1-ETO mutant, Lysine 43 mutated to Arginine |
| HA-AE9a | MigR1 | AE9a, Addgene plasmid #12433 |
| FLAG-ETO-W | pCMV | ETO wild type, full length, Addgene plasmid #12507 |
| FLAG-EZH1-W | pCMV6-entry | EZH1 wild type, full length, From Origene, RC202367 |
| FLAG-EZH1-ΔSET | pCMV6 | EZH1 mutant, without SET domain |
| FLAG-EZH1-ΔSANT | pCMV6 | EZH1 mutant, without SANT and SET domains |
| FLAG-EZH1-ΔWD | pCMV6 | EZH1 mutant, without WD, SANT and SET domains |
| His-EZH1-ΔSET | pcDNA4.0 | EZH1 mutant, without SET domain |
| His-EZH1-W | pcDNA4.0 | EZH1 wild type |
| His-EZH1-WD | pET41aHT | EZH1 mutant, only WD domain |
| His-AE-NHR1 | pET41aHT | AML1-ETO mutant, only NHR1 domain |
| GST-EZH1-WD | pGEX-GST | EZH1 mutant, only WD domain |
| GST-AE-NHR1 | pGEX-GST | AML1-ETO mutant, only NHR1 domain |
| GST-AE-W | pGEX-5X-1 | AML1-ETO wild type, residue 23-443 |
| GST -AE-K43R | pGEX-5X-1 | AML1-ETO mutant, Lysine 24 mutated to Arginine |
| GST-EZH1-ΔSET | pGEX-5X-1 | EZH1 mutant, without SET domain |
| GST-EZH1-W | pGEX-5X-1 | EZH1 wild type, full length |
| GST-EZH1-SET | pGEX-5X-1 | EZH1 mutant, only SET domain |
| V2LMM_102131-ep300 | pGIPZ | shRNA for p300, from BMGC RNAi |

| | | |
|---|---|---|
| V3LHS_331296-ep300 | pGIPZ | shRNA for p300, from BMGC RNAi |
| V3LHS_331297-ep300 | pGIPZ | shRNA for p300, from BMGC RNAi |
| V2LHS_151492-EZH1 | pGIPZ | shRNA for EZH1, from BMGC RNAi |
| V2LHS_151493-EZH1 | pGIPZ | shRNA for EZH1, from BMGC RNAi |
| V2LHS_151495-EZH1 | pGIPZ | shRNA for EZH1, from BMGC RNAi |
| TRCN0000002439-EZH1 | pLKO.1 | shRNA for EZH1, from BMGC RNAi |
| TRCN0000002440-EZH1 | pLKO.1 | shRNA for EZH1, from BMGC RNAi |
| TRCN0000002441-EZH1 | pLKO.1 | shRNA for EZH1, from BMGC RNAi |
| pSMP-EZH1_2 | pSMP | shRNA for EZH1, Addgene plasmid # 36360 |
| pSMP-EZH1_1 | pSMP | shRNA for EZH1, Addgene plasmid # 36359 |
| pSMP-EZH1_3 | pSMP | shRNA for EZH1, Addgene plasmid # 36361 |
| FLAG-p300-W | pcDNA4.0 | p300 wild type, full length |
| HA-Ezh2-W | pCMV | Ezh2 wild type, full length, Addgene plasmid # 24230 |
| FLAG-Ezh2-W | LZRS | Ezh2 wild type, full length, Addgene plasmid # 26111 |
| V2LHS_256397-SUZ12 | pGIPZ | shRNA for SUZ12, from BMGC RNAi |
| V2LHS_74301-SUZ12 | pGIPZ | shRNA for SUZ12, from BMGC RNAi |
| V2LHS_23172-EED | pGIPZ | shRNA for EED, from BMGC RNAi |
| V2LHS_23174-EED | pGIPZ | shRNA for EED, from BMGC RNAi |
| pCMV5-AML1 | pCMV5 | AML1 wild type, full length, Addgene plasmid #12426 |
| AE L301H | pUHD | AML1-ETO mutant, Lysine 301 mutated to Histidine |
| AE-N305L | pUHD | AML1-ETO mutant, Asparagine 305 mutated to Leucine |
| AER297D | pUHD | AML1-ETO mutant, Arginine 297 mutated to Aspartic acid |
| EZH1L29H | pCMV6-entry | EZH1 mutant, Lysine 43 mutated to Histidine |
| AML1-K24R | pCMV5 | AML1 mutant, Lysine 24 mutated to Arginine |
| AML1-K43R | pCMV5 | AML1 mutant, Lysine 43 mutated to Arginine |
| FLT3-ITD | pMSCV | FLT3-ITD, Addgene plasmid #74499 |

**Supplementary Methods and Parameters for Proteomic Analysis**

**Detailed description of the database search parameters and acceptance criteria**

All relevant parameters of the LC-MS experiment are described in Methods part (Page 24, Paragraph 1, Methods Part). T.T. Lam, K. Wilczak and J. Kanyo at the MS & Proteomics Resource at Yale University worked for the high-resolution mass spectrometry data. Peptides identification criteria are set at MASCOT Expectation level of 0.05 (e.g. 95% confidence) for all peptides (http://www.matrixscience.com/help/scoring_help.html). MASCOT Search Engine (Matrix Science, v. 2.4), Protein database: SWISSPROT_2014_August. With a minimum of 2 or more peptides to be present for protein ID presense. The variable modifications were Oxidation (M), propionamide (C), Methyl (K), Dimethyl (K), Trimethyl (K). The peptide mass tolerance was 20 ppm and the fragment mass tolerance was 0.5 Da (Fig. 1).

YPED

**Sample: K-1 MASCOT 1::Dou_AML1ETO_v1.fasta 2::SwissProt_2014_08.fasta**

**Mascot Search Parameters**

| | |
|---|---|
| **Cleavage Enzyme** | Trypsin/P |
| **Maximum Number of Missed Cleavages** | 2 |
| **Fixed Modifications** | |
| **Variable Modifications** | Oxidation (M), Propionamide (C), Methyl (K), Dimethyl (K), Trimethyl (K) |
| **Peptide Mass Tolerance** | 20 ppm |
| **Fragment Mass Tolerance** | 0.5 Da |
| **Monoisotopic or Average** | Monoisotopic |

For questions or comments contact MS & Proteomics Resource
Updated 30 Apr 2018 15:50

**Figure 1.** Database search parameters and acceptance criteria used for peptide identification.

**Proteomics Orbitrap Methods**

Creator: Orbitrap Elite
Last modified: 9/11/2014 by Orbitrap Elite

MS Run Time (min): 140.00

Sequence override of method parameters not enabled.

Divert Valve: not used during run

Contact Closure: not used during run

Syringe Pump: not used during run

MS Detector Settings:

Real-time modifications to method disabled

Stepped collision energy not enabled

Additional Microscans:

| | | |
|---|---|---|
| MS2 | 0 | 0 |
| MS3 | 0 | 0 |
| MS4 | 0 | 0 |
| MS5 | 0 | 0 |

MS6 0 0
MS7 0 0
MS8 0 0
MS9 0 0
MS10 0 0

Experiment Type: Nth Order Double Play

Tune Method: 082214NS

Scan Event Details:
1: FTMS + p norm o(300.0-2000.0)
CV = 0.0V
2: ITMS + c norm Dep MS/MS Most intense ion from (1)
Activation Type: CID
Min. Signal Required: 500.0
Isolation Width: 2.00
Normalized Coll. Energy: 35.0
Default Charge State: 2
Activation Q: 0.250
Activation Time: 10.000
CV = 0.0V
Scan Event 2 repeated for top 15 peaks.

Lock Masses:
Pos List Name: N/A
Source: API Source
Mass List: 445.120024
Neg List Name: N/A
Source: API Source
Mass List: (none)

Data Dependent Settings:
Use separate polarity settings disabled
Parent Mass List: (none)
Reject Mass List: (none)
Neutral Loss Mass List: (none)
Product Mass List: (none)
Neutral loss in top: 3
Product in top: 3
Most intense if no parent masses found not enabled
Add/subtract mass not enabled
FT master scan preview mode enabled
Charge state screening enabled
Charge state dependent ETD time not enabled
Monoisotopic precursor selection enabled
Charge state rejection enabled
Unassigned charge states : rejected
Charge state 1 : rejected
Charge state 2 : not rejected
Charge state 3 : not rejected

Charge states 4+ : not rejected
Chromatography mode is disabled

Global Data Dependent Settings:
Predict ion injection time enabled
Use global parent and reject mass lists not enabled
Exclude parent mass from data dependent selection not enabled
Exclusion mass width relative to mass
Exclusion mass width relative to low (ppm): 10.000
Exclusion mass width relative to high (ppm): 10.000
Parent mass width by mass
Parent mass width low: 0.50000
Parent mass width high: 0.50000
Reject mass width by mass
Reject mass width low: 0.50000
Reject mass width high: 0.50000
Zoom/UltraZoom scan mass width by mass
Zoom/UltraZoom scan mass width low: 5.00
Zoom/UltraZoom scan mass width high: 5.00
FT SIM scan mass width low: 5.00
FT SIM scan mass width high: 5.00
Neutral Loss candidates processed by decreasing intensity
Neutral Loss mass width by mass
Neutral Loss mass width low: 0.50000
Neutral Loss mass width high: 0.50000
Product candidates processed by decreasing intensity
Product mass width by mass
Product mass width low: 0.50000
Product mass width high: 0.50000
MS mass range: 0.00-1000000.00
MSn mass range by mass
MSn mass range: 0.00-1000000.00
Use m/z values as masses not enabled
Analog UV data dep. not enabled
Dynamic exclusion enabled
Repeat Count: 1
Repeat Duration: 30.00
Exclusion List Size: 500
Exclusion Duration: 60.00
Exclusion mass width relative to mass
Exclusion mass width relative to low (ppm): 10.000
Exclusion mass width relative to high (ppm): 10.000
Expiration Count: 2
Expiration S/N Threshold: 2.0
Isotopic data dependence not enabled

---

Chromatography:

Method

RunTime: 140.00
nAcquitySMMethod
RunTime: 1.0
Comment
Mode: ModeSequential_0
LoopOption: LoopOptionPartial_1
LoopOffline: LoopOfflineDisable_0
WeakWashSolvent
WeakWashVolume: 1200
StrongWashSolvent
StrongWashVolume: 400
ColumnTemperature: 38.0
ColumnTemperatureLimit: 5.0
SampleTemperature: 4.0
SampleTemperatureLimit: 5
Overfill: CustomDisable_-1
ColumnReequilibration: 1.0
DrawRate: DrawRateMedium_1
CustomRate: CustomDisable_-1
Illumination: IlluminationOff_0
NeedleDepth: 0.7
PreAspirateAir: CustomDisable_-1
PostAspirateAir: CustomDisable_-1
ColumnTemperatureDataEnable: false
AmbientTemperatureDataEnable: false
SampleTemperatureDataEnable: false
ElevatorTemperatureDataEnable: false
SamplePressureDataEnable: false
Switch1: InitialSwitchState_5
Switch2: InitialSwitchState_5
Switch3: InitialSwitchState_5
Switch4: InitialSwitchState_5
Switch5: InitialSwitchState_5
Switch6: InitialSwitchState_5
HighPressureLimit: 5000
LowPressureLimit: 0
ChartOut: ChartOut_1
SampleTempAlarmEnable: false
ColumnTempAlarmEnable: false
RunEvents: true
EventTable
Function: FunctionNone
ColumnManagerPresent: false
ColumnManager
ValvePosition: ValvePosition_1
SetColumnTemperature: HeaterOff_-1
EquilibrationTime: 0.1
EnableColumnTempDataChannel: false
EnableAlarmBand: true
SetAlarmBandDegC: 5.0
Switch1: InitialSwitchState_5

    Switch2: InitialSwitchState_5
    Switch3: InitialSwitchState_5
    Switch4: InitialSwitchState_5
    RunEvents: false
  NeedleOverfillFlush: CustomDisable_-1
  SampleLoop: 5.00
  ApplicationMode: ApplicationMode_1
  InjFromTrap: false
  IsTrizaic: false
  TrizaicChipCoolDownMins: 2.0
  DisableFlowRamps: DisableFlowRamps_0
nAcquityBSMMethod
  Scale: ScaleNano_1
  ApplicationMode: ApplicationMode_2
  PumpRole: PumpType_1
  HTM: false
  RunTime: 30.0
  FlowSourceA: 1
  FlowSourceB: 1
  SolventNameA: Water
  SolventNameB: Acetonitrile
  SealWashPeriod: 30.0
  InitialSwitch1: SwitchNoChange_4
  InitialSwitch2: SwitchNoChange_4
  InitialSwitch3: SwitchNoChange_4
  VentValve: VentValveSystem_2
  Analog1: AnalogPressure_2
  Analog2: AnalogB_4
  RunEvents: true
  PostTrapCondition: false
  GradientTable
    GradientRow
      Time: Initial
      Flow: 0.300
      CompositionA: 97.0
      CompositionB: 3.0
      Curve
    GradientRow
      Time: 1.00
      Flow: 0.300
      CompositionA: 97.0
      CompositionB: 3.0
      Curve: 6
    GradientRow
      Time: 70.00
      Flow: 0.300
      CompositionA: 70.0
      CompositionB: 30.0
      Curve: 6
    GradientRow
      Time: 90.00

```
      Flow: 0.300
      CompositionA: 50.0
      CompositionB: 50.0
      Curve: 6
   GradientRow
      Time: 95.00
      Flow: 0.300
      CompositionA: 15.0
      CompositionB: 85.0
      Curve: 6
   GradientRow
      Time: 100.00
      Flow: 0.300
      CompositionA: 15.0
      CompositionB: 85.0
      Curve: 6
   GradientRow
      Time: 101.00
      Flow: 0.300
      CompositionA: 97.0
      CompositionB: 3.0
      Curve: 6
EventTable
FlowRate: 0.3
ElutionCompositionA: 97.0
ElutionCompositionB: 3.0
LowPressureLimit: 0
HighPressureLimit: 9500
NanoParameters
   SampleLoadingTime: 3.00
   BSMTrappingFlowRate: 5.000
   BSMTrappingCompositionA: 97.0
   BSMTrappingCompositionB: 3.0
   TrappingLowPressureLimit: 0
   TrappingHighPressureLimit: 9500
   VariableFlowFactor: 0.10
   TrappingMode: TrappingMode_2
   EnablePeakParking: false
   FlowRateADataEnable: false
   FlowRateBDataEnable: false
   TemperatureADataEnable: false
   TemperatureBDataEnable: false
SolventTypeA: SolventType_2
SolventTypeB: SolventType_3
Comment
AuxPump: false
DegasserEnable: true
SystemPressureDataEnable: false
FlowRateDataEnable: false
PercentADataEnable: false
PercentBDataEnable: false
```

LeftAPressureDataEnable: false
RightAPressureDataEnable: false
LeftBPressureDataEnable: false
RightBPressureDataEnable: false
DegasserPressureDataEnable: false
AuxPumpAParameters
  AuxPumpRole: Auxiliary
  AuxSolventName: Water
  AuxSolventType: SolventType_2
  AuxFlow: 0.000
  AuxFlowSource: 1
  AuxLowPressureLimit: 0
  AuxHighPressureLimit: 10000
  AuxPressureDataEnable: false
  AuxFlowRateDataEnable: false
AuxPumpBParameters
  AuxPumpRole: Lock Mass
  AuxSolventName: Water
  AuxSolventType: SolventType_2
  AuxFlow: 0.000
  AuxFlowSource: 1
  AuxLowPressureLimit: 0
  AuxHighPressureLimit: 10000
  AuxPressureDataEnable: false
  AuxFlowRateDataEnable: false
