# Supplementary material for: Protein lysine 43 methylation by EZH1 promotes AML1-ETO transcriptional repression in leukemia
Source: Nat Commun. 2019 Nov 7;10:5051. doi: 10.1038/s41467-019-12960-6 (PMC6838331; doi:10.1038/s41467-019-12960-6)
Supplement: Supplementary file 2 — Description of Additional Supplementary Files [file 41467_2019_12960_MOESM2_ESM.pdf]

Description of Additional Supplementary Files

File Name: Supplementary Data 1
Description: Upregulated genes with AML1-ETO binding sites regulated by AML1- ETO in Kasumi-1 cells.

File Name: Supplementary Data 2
Description: Downregulated genes with AML1-ETO binding sites regulated by AML1- ETO in Kasumi-1 cells.

File Name: Supplementary Data 3
Description: Downregulated genes with methylated-AEK43 binding sites regulated by methylated-AEK43 in Kasumi-1 cells.

File Name: Supplementary Data 4
Description: Upregulated genes with acetylated-AEK43 binding sites regulated by acetylated-AEK43 in Kasumi-1 cells.
