# Supplementary material for: Protein lysine 43 methylation by EZH1 promotes AML1-ETO transcriptional repression in leukemia
Source: Nat Commun. 2019 Nov 7;10:5051. doi: 10.1038/s41467-019-12960-6 (PMC6838331; doi:10.1038/s41467-019-12960-6)
Supplement: Supplementary file 7 — Reporting Summary [file 41467_2019_12960_MOESM7_ESM.pdf]

nature research

Corresponding author(s): Li Yu; Shujun Liu

Reporting Summary

Nature Research wishes to improve the reproducibility of the work that we publish. This form provides structure for consistency and transparency in reporting. For further information on Nature Research policies, see Authors & Referees and the Editorial Policy Checklist.

Statistical parameters

When statistical analyses are reported, confirm that the following items are present in the relevant location (e.g. figure legend, table legend, main text, or Methods section).

n/a	Confirmed	
☐	☒	The exact sample size (n) for each experimental group/condition, given as a discrete number and unit of measurement
☐	☒	An indication of whether measurements were taken from distinct samples or whether the same sample was measured repeatedly
☐	☒	The statistical test(s) used AND whether they are one- or two-sided<br>*Only common tests should be described solely by name; describe more complex techniques in the Methods section.*
☐	☒	A description of all covariates tested
☐	☒	A description of any assumptions or corrections, such as tests of normality and adjustment for multiple comparisons
☐	☒	A full description of the statistics including central tendency (e.g. means) or other basic estimates (e.g. regression coefficient) AND variation (e.g. standard deviation) or associated estimates of uncertainty (e.g. confidence intervals)
☒	☐	For null hypothesis testing, the test statistic (e.g. F, t, r) with confidence intervals, effect sizes, degrees of freedom and P value noted<br>*Give P values as exact values whenever suitable.*
☒	☐	For Bayesian analysis, information on the choice of priors and Markov chain Monte Carlo settings
☐	☒	For hierarchical and complex designs, identification of the appropriate level for tests and full reporting of outcomes
☐	☒	Estimates of effect sizes (e.g. Cohen's d, Pearson's r), indicating how they were calculated
☐	☒	Clearly defined error bars<br>*State explicitly what error bars represent (e.g. SD, SE, CI)*

Our web collection on statistics for biologists may be useful.

Software and code

Policy information about availability of computer code

Data collection	SPSS 15.0 software
Data analysis	No code

For manuscripts utilizing custom algorithms or software that are central to the research but not yet described in published literature, software must be made available to editors/reviewers upon request. We strongly encourage code deposition in a community repository (e.g. GitHub). See the Nature Research guidelines for submitting code & software for further information.

Data

Policy information about availability of data

All manuscripts must include a data availability statement. This statement should provide the following information, where applicable:

- Accession codes, unique identifiers, or web links for publicly available datasets
- A list of figures that have associated raw data
- A description of any restrictions on data availability

Data will be available with no restriction; Figure 5 is associated with raw data; No accession codes.

April 2018

Field-specific reporting

Please select the best fit for your research. If you are not sure, read the appropriate sections before making your selection.

☒ Life sciences ☐ Behavioural & social sciences ☐ Ecological, evolutionary & environmental sciences

For a reference copy of the document with all sections, see nature.com/authors/policies/ReportingSummary-flat.pdf

Life sciences study design

All studies must disclose on these points even when the disclosure is negative.

Sample size	The sample sizes for each study were chosen to be sufficient to allow statistical analysis of the outcomes of the experimental versus control of the studies based on literature documentation of similar well-characterized experiments. All analyses were performed using the GraphPad Prism 5 Software.
Data exclusions	No samples or animals were excluded from the analysis. All criteria were pre-established.
Replication	In vitro experiments, such as qPCR, Western blotting, cell proliferation assays, dotblotting, clonogenic assays etc. were routinely repeated three times unless indicated otherwise in Figure legends or main text.
Randomization	No randomization was used in our studies. Please see Method, statistical analysis section.
Blinding	No blinding for all experiments.

Reporting for specific materials, systems and methods

Materials & experimental systems

n/a	Involved in the study	
☐	☒	Unique biological materials
☐	☒	Antibodies
☐	☒	Eukaryotic cell lines
☒	☐	Palaeontology
☐	☒	Animals and other organisms
☐	☒	Human research participants

Methods

n/a	Involved in the study	
☐	☒	ChIP-seq
☐	☒	Flow cytometry
☒	☐	MRI-based neuroimaging

Unique biological materials

Policy information about availability of materials

Obtaining unique materials	No restrictions on availability of unique materials.

Antibodies

Antibodies used	The information for all antibodies used in our studies is described in Material Section and Supplementry Table 5
Validation	Validation of the purchased antibodies are described in the website of corresponding vendors; The customized polyclonal antibodies specific for the acetylated and mono-methylated AML1 at K43 (anti-acetylated K43-AML1 and anti-methylated K43-AML1) were validated by the PTM Biolab Inc, who produced these two antibodies.

Eukaryotic cell lines

Policy information about cell lines

Cell line source(s)	American Type Culture Collection; University of Rome, Italy (SKNO-1 and SKNO-1siAE).
Authentication	Cell lines were newly purchased with no further authentication. We froze initial cell line stocks and used early passages of cells (< 3 months in culture) in all experiments.

Mycoplasma contamination	Cell lines were newly purchased with no further testing for mycoplasma.
Commonly misidentified lines (See ICLAC register)	No cell line used in this paper is listed in the database of commonly misidentified cell lines maintained by ICLAC.

Animals and other organisms

Policy information about studies involving animals; ARRIVE guidelines recommended for reporting animal research

Laboratory animals	The information for all animals used in our studies are shown in Material and Method section. All animal experiments were approved by the Institutional Animal Care and Use Committees of the University of Minnesota and the Chinese PLA General Hospital, were in accordance with the U.S. National Institutes of Health (NIH) Guide for Care and Use of Laboratory Animals.
Wild animals	No wild animals were used.
Field-collected samples	The studies did not involve samples collected in the field.

Human research participants

Policy information about studies involving human research participants

Population characteristics	Characterization of patients are shown in main manuscript, page 32; Supplementary Table 1, Supplementary Table 2, and Supplementary Table 3 in Supplementary material, page 18-20.
Recruitment	No new patients were recruited specifically for this study.

ChIP-seq

Data deposition

☒ Confirm that both raw and final processed data have been deposited in a public database such as GEO.

☒ Confirm that you have deposited or provided access to graph files (e.g. BED files) for the called peaks.

Data access links<br>*May remain private before publication.*	All gene expression microarray data used in this manuscript are deposited in Gene Expression Omnibus (GEO) under the accession number GSE74237, the RNA-seq data in the Bioproject under the BioProject ID 397611, the ChIP-seq data in the Bioproject under the BioProject ID PRJNA298516 and 397609.<br><br>a) SRA sequencing (Bioproject):<br>ftp://ftp-trace.ncbi.nlm.nih.gov/sra/review/SRP115595_20171017_154636_2726e05d1c01c63<br>b0742fdbb3d89c0bc<br>Reviewer / collaborator link to metadata (SRP115976):<br>trace.ncbi.nlm.nih.gov/sra/review/SRP115976_20171017_154744_b1659515b9d1a59ebbc790e01084a8f0<br>Reviewer / collaborator link to metadata (SRP064831):<br>ftp://ftp-trace.ncbi.nlm.nih.gov/sra/review/SRP064831_20171017_154953_2726e05d1c01c63<br>b0742fdbb3d89c0bc).
Files in database submission	8 RNA profiles name: SKNO-1 con, SKNO-1 siAE, SKNO-1 con, SKNO-1 siEZH1; Kasumi-1 siAE Mrna 8-26-16, Kasumi-1 Mrna 8-25-16, Kasumi-1 Mrna 9-1-16, Kasumi-1 siAE 8-31-16.<br>12 ChIP seq file name: aceAML1-ETOK43, EZH1, aceAML1-ETOK43, P300, input, IgG. Each name has duplicate files.
Genome browser session (e.g. UCSC)	*Provide a link to an anonymized genome browser session for "Initial submission" and "Revised version" documents only, to enable peer review. Write "no longer applicable" for "Final submission" documents.*

Methodology

Replicates	Dubplicate
Sequencing depth	ChIP-seq reads of single-end were sequencd in length of 50. The average number of reads of each sample were 40 million.
Antibodies	Anti-AML1-ETO specific antibody, anti-EZH1, anti-p300, anti-acetylated AML1-ETO K43, and anti-methylated AML1-ETO K43.
Peak calling parameters	Mapping parameters: bowtie2 -N 1 -p 4 -x GenomeIndex | samtools view -q 20 -bS -o BamFile.<br>Peak calling parameters: macs14 -t IP.sort.bam -c input.sort.bam -f BAM -g GenomeSize -w -p 1e-5.
Data quality	Peaks were called by MACS using default parameters but -p 1e-5, which produced thousands enrichment regions.
Software	To avoid alignment error, raw reads were filtered to produce cleaned reads by using in-house script. Clean reads were mapped to reference genome by using software of Bowtie2 and uniquely mapped reads were used to call peak. MACS was used to identify regions of ChIP enrichment regions.

Flow Cytometry

Plots

Confirm that:

- [ ] The axis labels state the marker and fluorochrome used (e.g. CD4-FITC).
- [x] The axis scales are clearly visible. Include numbers along axes only for bottom left plot of group (a 'group' is an analysis of identical markers).
- [ ] All plots are contour plots with outliers or pseudocolor plots.
- [x] A numerical value for number of cells or percentage (with statistics) is provided.

Methodology

Sample preparation	Suspended cells were washed once with cold PBS and fixed in 70% Ethanol overnight at -20C. After fixation cells were washed with cold PBS. The cells were incubated with RNase A (200 ug/ml) and propidium Iodidie (20 ug/ml) for 30 minutes in the dark at room temperature.
Instrument	FACSCalibur manufactured by Becton Dickinson.
Software	Data were collected with Cell Quest Pro V. 6.0 by Becton Dickinson. Data were analyzed with ModFit LT for Mac V. 4.1.7.
Cell population abundance	No sorting was done on these samples.
Gating strategy	Intact cells were gated in the FSC/SSC plot to exclude debris. Gated cells were plotted on an FL2-width vsl FL2 area dot plot. A gate was drawn to exclude doublets.

- [x] Tick this box to confirm that a figure exemplifying the gating strategy is provided in the Supplementary Information.
