# Supplementary material for: Protein lysine 43 methylation by EZH1 promotes AML1-ETO transcriptional repression in leukemia
Source: Nat Commun. 2019 Nov 7;10:5051. doi: 10.1038/s41467-019-12960-6 (PMC6838331; doi:10.1038/s41467-019-12960-6)
Supplement: Supplementary file 8 — Source Data [file 41467_2019_12960_MOESM8_ESM.zip › 185902_2_related_ms_4095509_py5b2z.pdf]

Figure 1

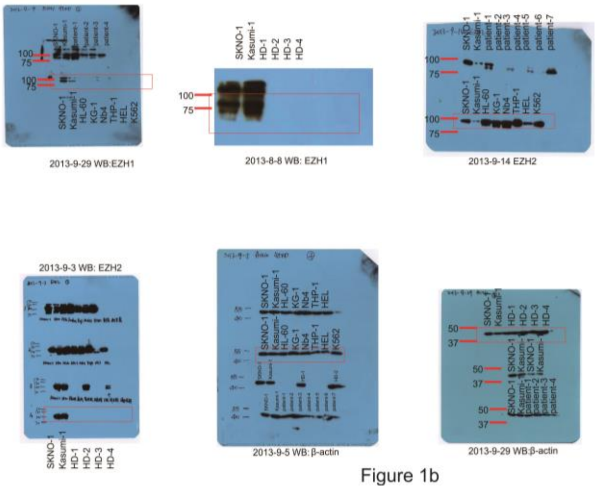

Figure 1b

Figure 2

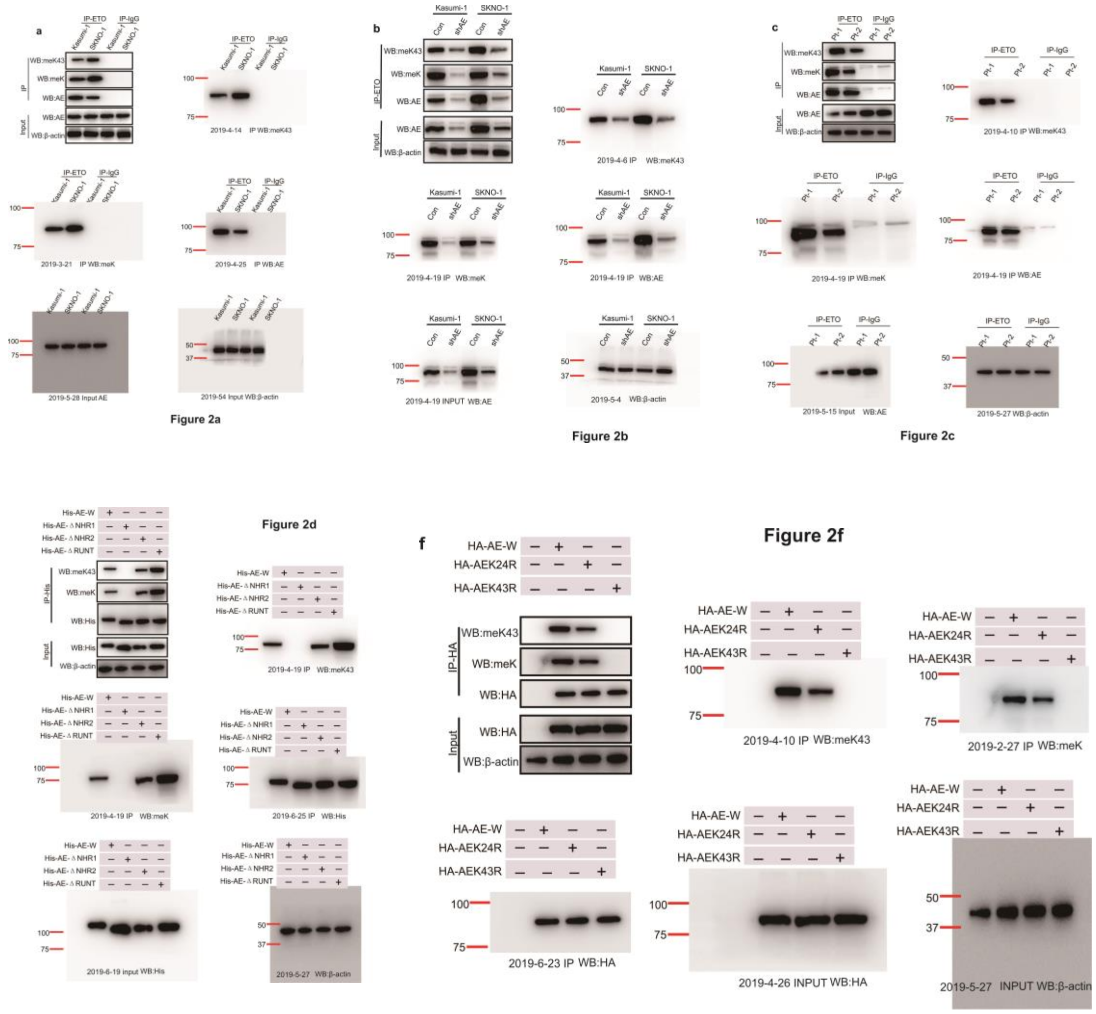

Figure 2a
Figure 2b
Figure 2c
Figure 2d
Figure 2f
2019-4-10 IP WB:meK43
2019-2-27 IP WB:meK
2019-6-23 IP WB:HA
2019-4-26 INPUT WB:HA
2019-5-27 INPUT WB:β-actin

Figure 3

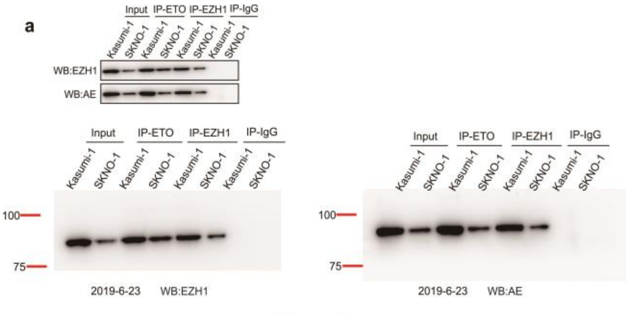

Figure 3a

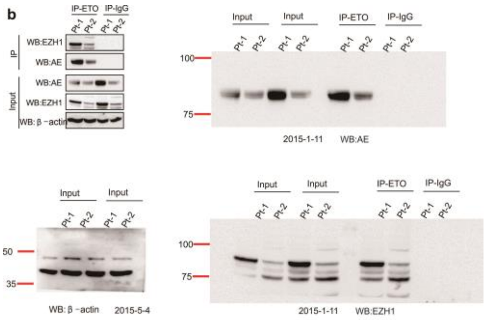

Figure 3b

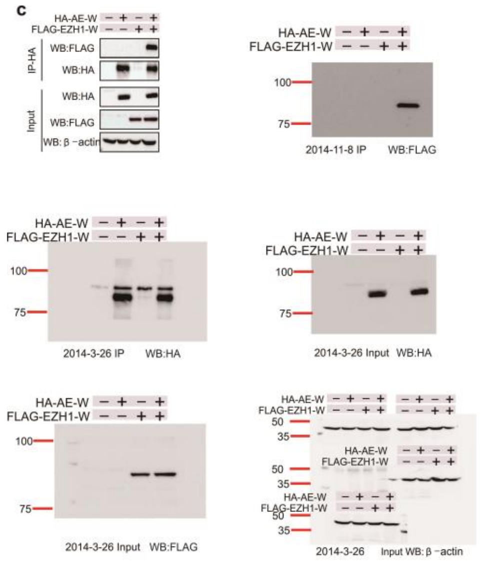

Figure 3c

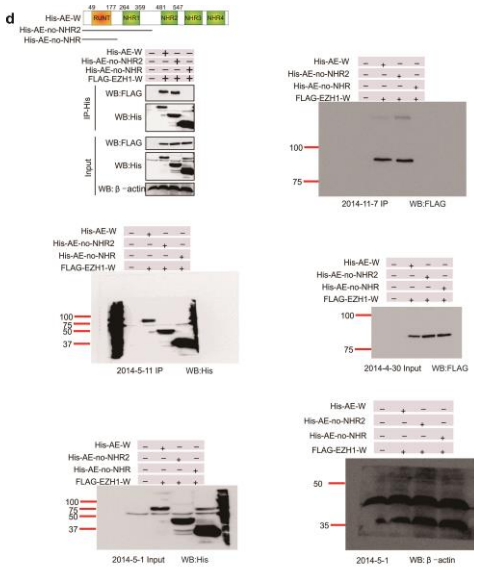

Figure 3d

Figure 3e

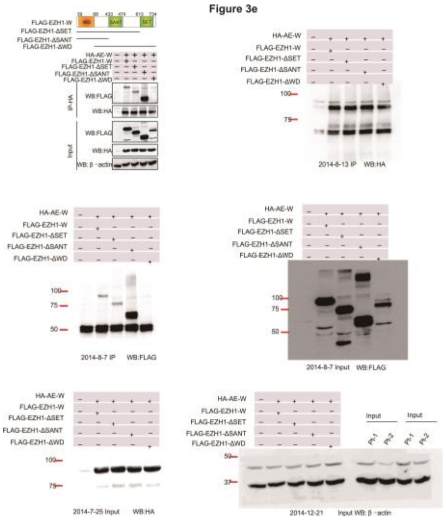

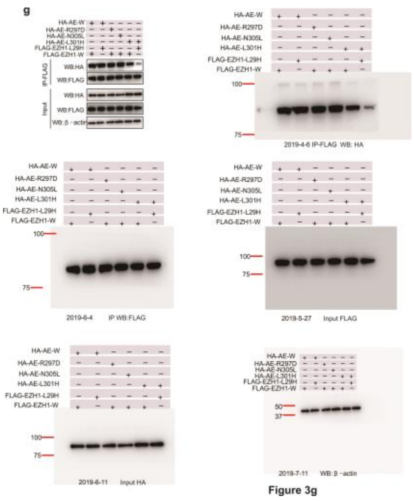

Figure 3g

Figure 4

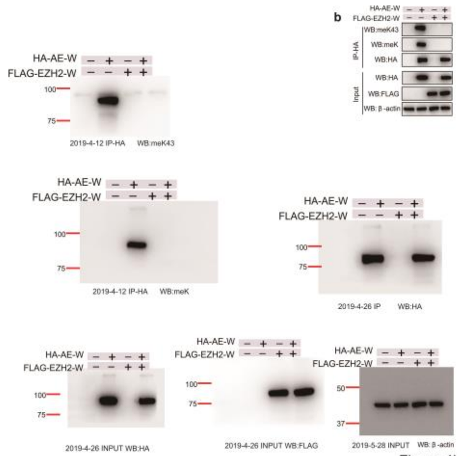

Figure 4b

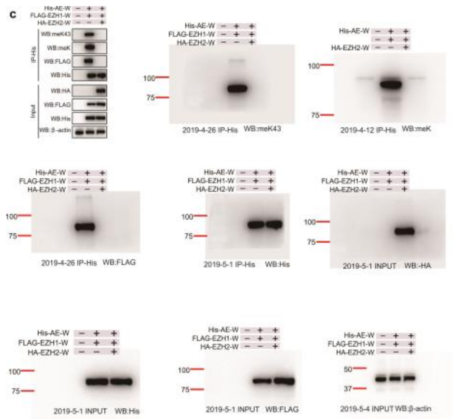

Figure 4c

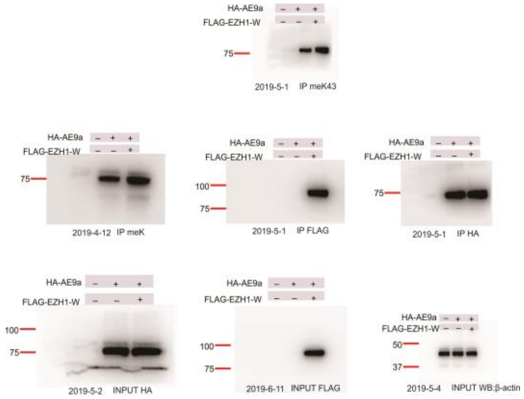

Figure 4e

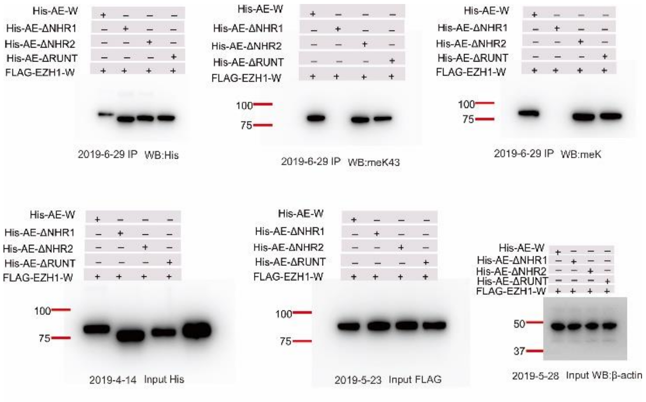

Figure 4d

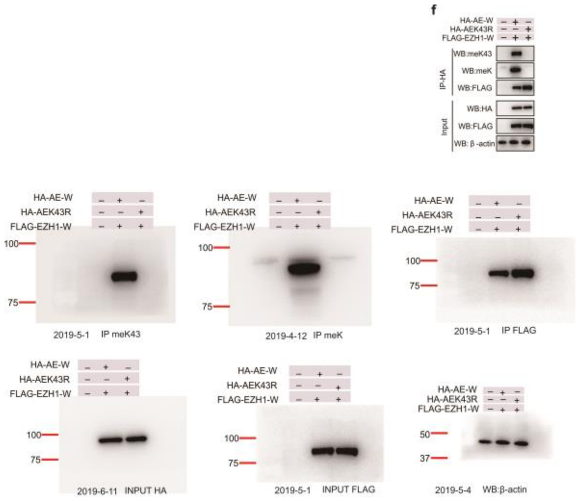

Figure 4f

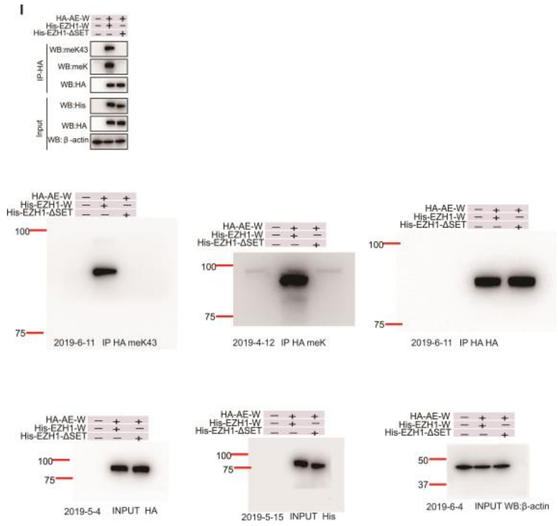

Figure 4i

Figure S1

Figure S1e

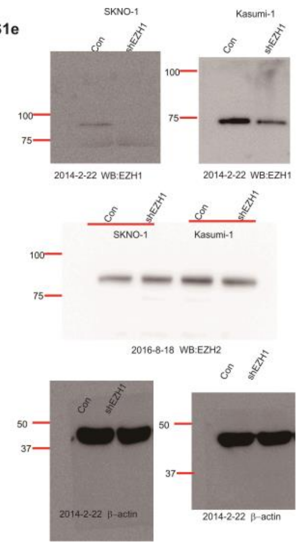

Figure S1j

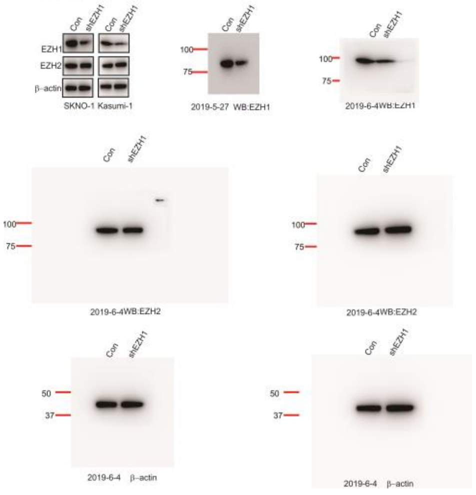

Figure S2

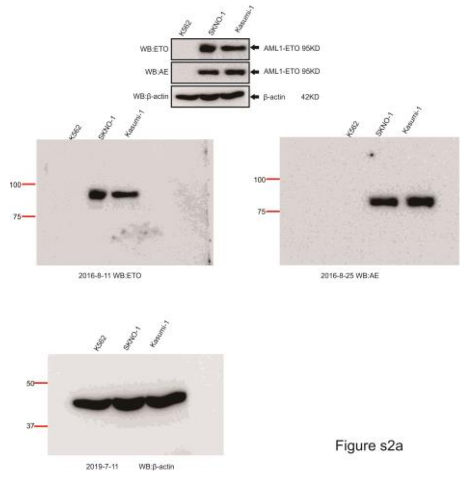

Figure s2a

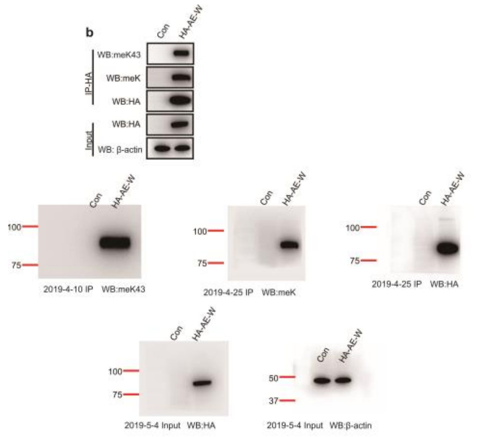

Figure s2b

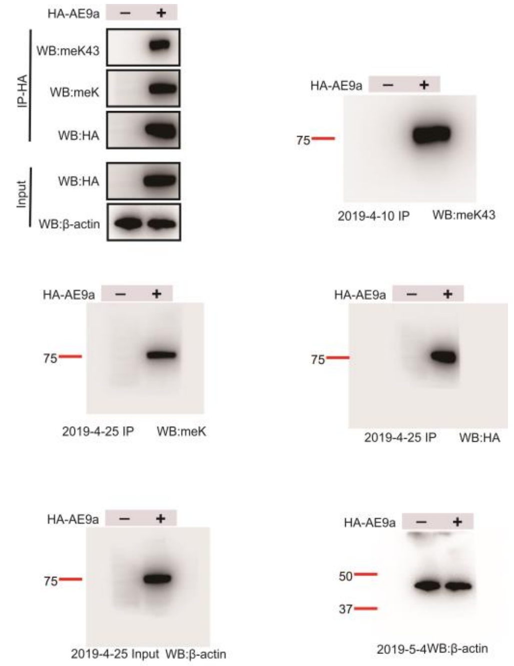

Figure S2c

Figure S3

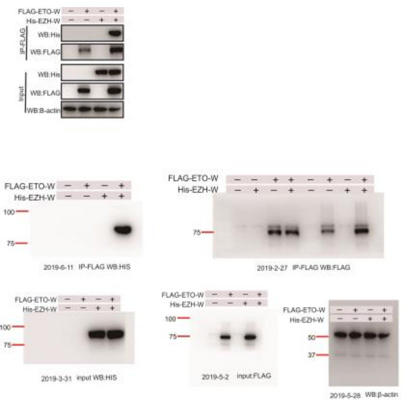

Figure S3

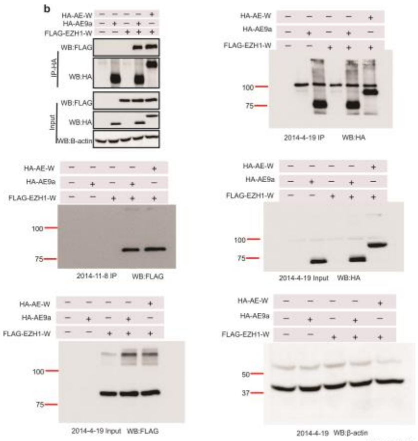

Figure S3b

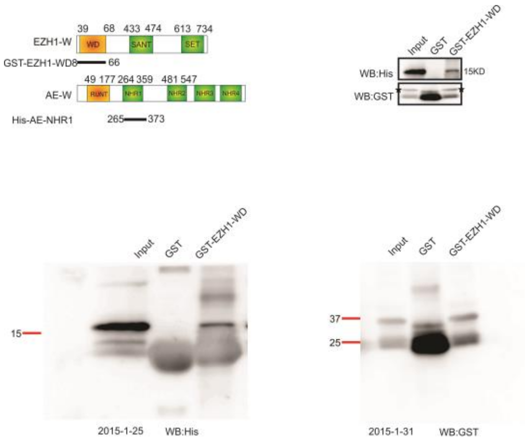

Figure S3d

Figure S4

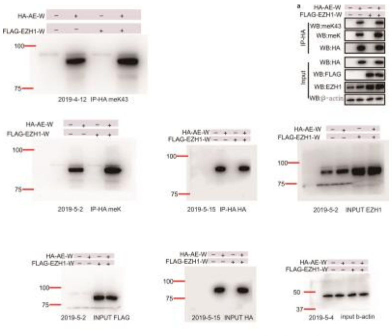

Figure S4a

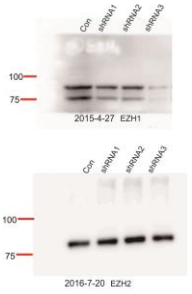

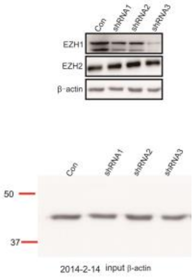

Figure S4b

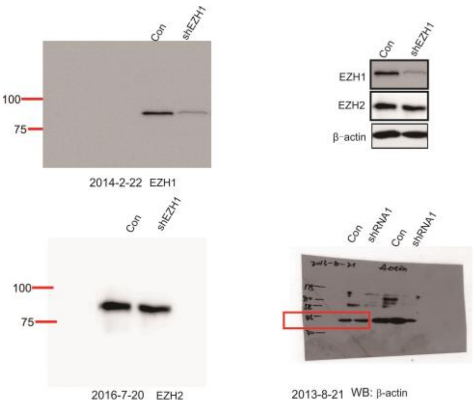

Figure S4c

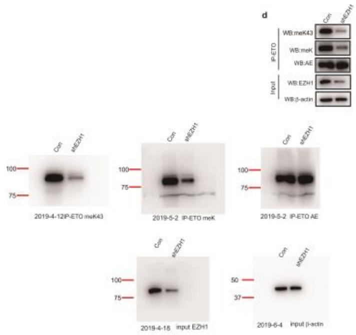

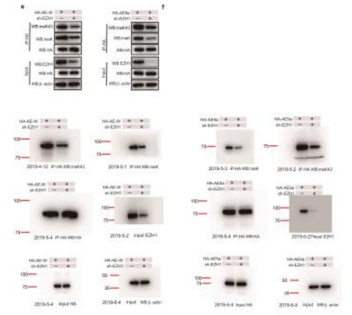

Figure S4e

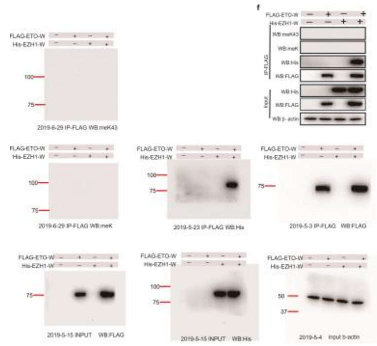

Figure S4f

Figure S4g

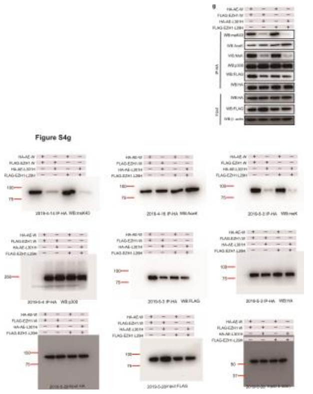

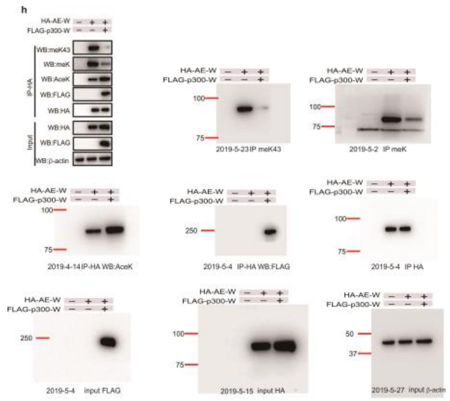

Figure S4h

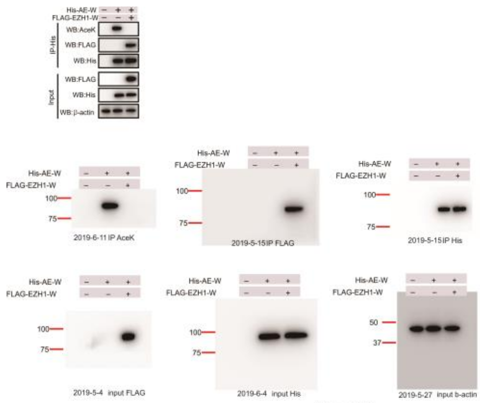

Figure S4i

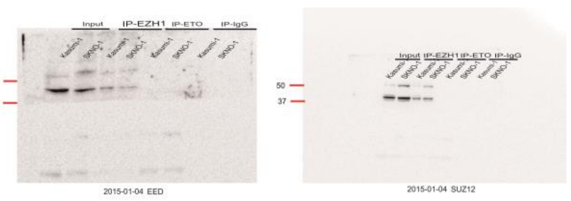

Figure S4j

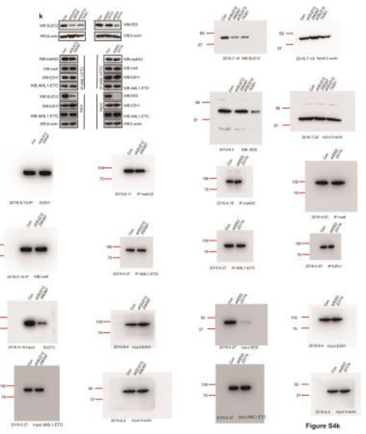

Figure S4k

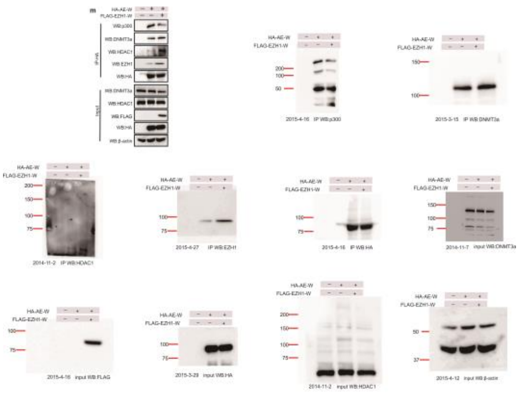

Figure S4m

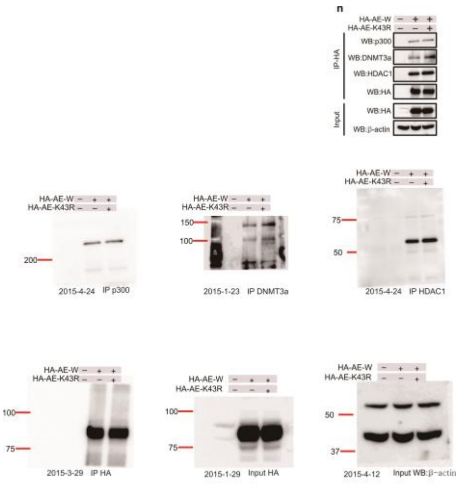

Figure S4n

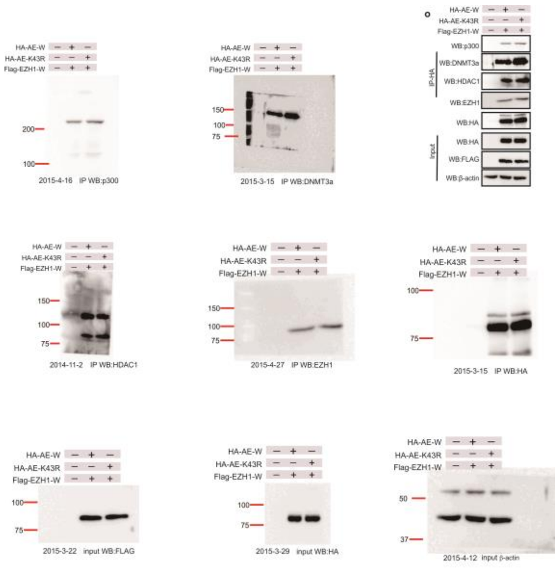

Figure S4o

Figure S5

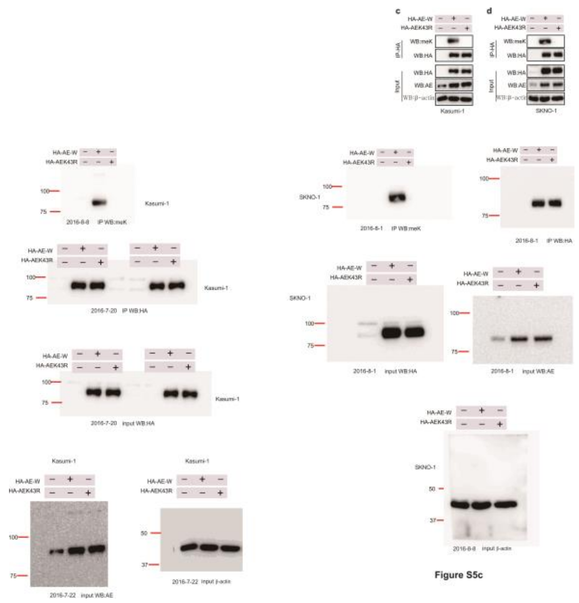

Figure S5c

Figure S6

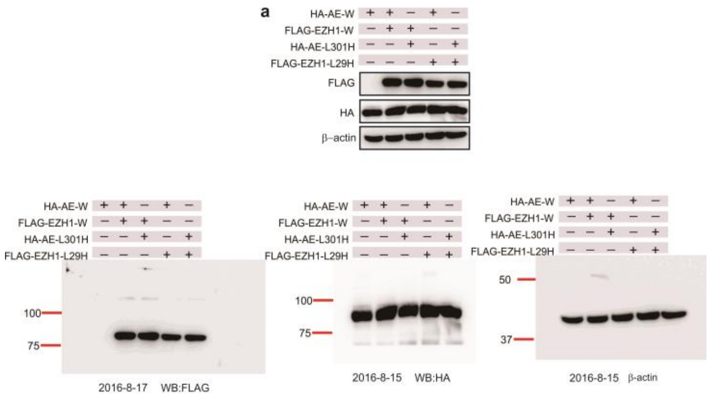

a
HA-AE-W
FLAG-EZH1-W
HA-AE-L301H
FLAG-EZH1-L29H
FLAG
HA
β-actin
100
75
2016-8-17 WB:FLAG
100
75
2016-8-15 WB:HA
50
37
2016-8-15 β-actin
